# Aging and Altered Gravity: A Cellular Perspective

**DOI:** 10.1096/fj.202402989R

**Published:** 2025-06-27

**Authors:** Sharon van Rijthoven, Jack J. W. A. van Loon

**Affiliations:** ^1^ Delft University of Technology Delft the Netherlands; ^2^ Department of Oral and Maxillofacial Surgery, Amsterdam Movement Sciences & Amsterdam Bone Center (ABC) Amsterdam UMC Location Vrije Universiteit Amsterdam & Academic Center for Dentistry Amsterdam (ACTA) Amsterdam the Netherlands; ^3^ European Space Agency (ESA), European Space Research and Technology Centre (ESTEC), TEC‐MMG Noordwijk the Netherlands

**Keywords:** agingaltered gravity, hypergravity, microgravity, senescence

## Abstract

The elderly and astronauts exhibit strikingly similar phenotypes. Although much research has addressed the comparison between these two groups at the level of the whole organism or organ level, like the musculoskeletal system, comparative studies at the cellular level remain limited. Therefore, this article aims to address this gap by conducting an extensive scoping review, comparing cell function and alterations with advanced age to those observed in altered gravity. The broad review spans different cell types and species, highlighting the generic nature of aging and its relationship to gravity. We identified 165 signs of aging at the cell level, deducted from leading aging papers, and grouped them into 11 themes: DNA and epigenetics, mitochondria, nucleus, immune system, protein and metabolism, lysosome and degradation, cell cycle, cytoskeleton, extracellular matrix (ECM), cell mechanics, and cell signaling. Following this classification, we conducted a comprehensive search using the databases Web of Science and PubMed to examine the behavior of these signs in altered gravity conditions. The results reveal that only 29% of the responses are similar in (simulated) microgravity compared to biological aging, while others show contrasting behavior, thereby highlighting the complexity of cellular responses in these conditions. However, the majority of the signs remain unexplored in altered gravity. Mechanotransduction emerges as a potential key player in the observed phenotypic resemblances between aging and microgravity. Since there still is quite a lack of knowledge of aging‐related effects on a cellular level in gravity‐related research, we recommend further gravity research on the many components making up the links that facilitate mechanotransduction, which can aid in understanding the origins of these shared phenotypes and could lead to new insights into age‐related and space‐induced health challenges.

## Graphical abstract

We propose that, unlike biological aging, where *Primary hallmarks* initiate damage, spaceflight may trigger a feedback loop between *Antagonistic* and *Integrative hallmarks*, driving accelerated aging. Returning to 1 g restores proper signaling and breaks the cycle. Though phenotypes appear similar at the cellular level, only 29% of studies show overlap between biological aging and (simulated) microgravity, while 32% report opposing or ambiguous effects.

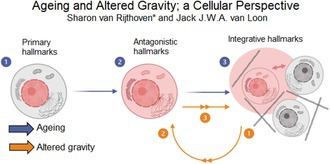

## 1 Introduction

Biological aging is a complex process that manifests itself in many, if not all, areas of the body and occurs on micro and macro scales in every life form. It is described as a gradual loss of physiological integrity, with increasing dysfunction and greater susceptibility to death [1]. Aging is often accompanied by age‐related diseases, which include neurodegenerative diseases like Alzheimer's and Parkinson's disease, cardiovascular diseases like heart failure and atherosclerosis, musculoskeletal disorders such as osteoarthritis and osteoporosis, immune system diseases, and cancer [2, 3]. It is hypothesized that the origin of aging lies in the time‐dependent accumulation of damage [1, 4, 5, 6]. Although anti‐aging drugs and therapies exist to counteract age‐associated diseases, they still come with many side effects and do not guarantee healthy aging [3].

Astronauts and the elderly seem to experience similar biological aging symptoms. From the well‐known similarities in osteoporosis and muscle atrophy, which are currently partly counteracted by excessive exercise, to more recent discoveries in neuro‐immuno‐endocrine‐metabolic interactions [7, 8, 9]. The comparison between aging and microgravity is not novel and has been discussed in many articles before [8, 9, 10, 11, 12, 13, 14]. It still remains uncertain what lies at the root of this resemblance. Many argue that gradually deconditioning as you age stems from a progressive unloading in exercise and reduced activities, while in microgravity the unloading is instantaneous, thereby resulting in accelerated aging symptoms [13]. Although a comparison is often made for higher‐level systems in the body, like tissue and organs, relatively few analyses have been made on a cellular level. Cell function seems to alter in microgravity, yet an extensive scoping review comparing cell function and alterations in altered gravity and with advanced age is absent in literature [15]. Therefore, our research question is:

*What are the differences and similarities between biological aging and altered gravity from a cellular perspective?*

To answer this question, we used the comprehensive works of López‐Otín et al. [1], Schmauck‐Medina et al. [16], Bajpai et al. [17], Phillip et al. [18], and Starodubtseva [19] to identify the signs of aging in cells. Although we break the problem down into small items, one must keep in mind that this is a simplification to approach the complex phenomenon of aging. In reality, many observations are interconnected. After obtaining the signs of aging of the five papers mentioned above, PubMed and Web of Science are used to search for gravity‐related research performed on these particular items. In our approach, we selected three types of altered gravity: hypergravity, simulated microgravity, and real microgravity, as we want to investigate the effect across the gravity spectrum. When comparing aging with altered gravity, we expect similar effects between aging and simulated or real microgravity, and opposite effect between aging and hypergravity based on the paradigm that gravity acts as a continuum [20, 21]. Furthermore, we make the distinction between simulated and real microgravity since even though these environments appear identical, they are not one and the same [20, 22, 23]. Real microgravity, or better near weightlessness, is a state of true free fall, whereas simulated microgravity often involves changing or randomizing the direction of the gravity vector relative to the sample (e.g., clinostat and RPM) [24]. This process can introduce additional forces, such as shear stress [25]. Other simulation methods rely on mechanical unloading, such as bed rest studies and hindlimb suspension [20, 22, 23]. The difference between real and simulated microgravity becomes apparent in studies that include both conditions. From Table S1 in the Supporting Information, we identified 15 studies that directly compared real and simulated microgravity within the same experimental setup. If the two environments were equivalent, outcomes should be consistent across both. However, only 5 of these studies reported strong resemblance [26, 27, 28, 29, 30], 4 showed partial resemblance [31, 32, 33, 34], and 6 showed no to little resemblance [35, 36, 37, 38, 39, 40]. While simulated microgravity remains a valuable and accessible ground‐based model, it is a suboptimal substitute for real microgravity and should therefore be treated as a distinct condition when interpreting results.

By investigating the above research question, we can get a first impression of whether gravity and aging exhibit comparable symptoms at the cellular level, mirroring their observed similarities in higher‐level systems, thereby aiding in understanding the origins of these shared phenotypes. Furthermore, it helps to decide whether research in altered gravity conditions can be used as a model for aging studies. In addition, insights into accelerated aging‐like effects in microgravity could aid the development of new drugs and devices to counteract the detrimental effects of space on future astronauts. Finally, a better understanding of the fundamentals of aging could lead to new medicines, therapies, and technologies in the medical field that promote healthy aging, which will improve the quality of life on Earth.

## 2 Material and Methods

### 2.1 Aging Signs

From the works on biological aging from López‐Otín et al. [1], Schmauck‐Medina et al. [16], Bajpai et al. [17], Phillip et al. [18], and Starodubtseva [19], we extracted 209 signs that alter during the process of aging or influence lifespan or longelivity. Although one has to be aware that longevity is not necessarily the same as increased aging [41]. These articles were selected for their comprehensive analysis of various cellular aging symptoms and their noticeable citation count. We assume, based on their findings, that these represent true hallmarks of aging, though we acknowledge that absolute certainty is unattainable. The papers cover a wide range of biological systems, from humans to flies and from primary cells to cancer cell lines. While this diversity may obscure some nuances, it facilitates the identification of general aging patterns. Some aging signs are derived from progeria models, which, despite their limitations, are commonly used in aging research due to shared characteristics with biological aging [42]. In the text we refer to the aging characteristics as “signs” or “items” The aging signs are not independent of one another and can interact. Separating them in single observations is a simplification used to approach the complex phenomenon of aging. Due to this intertwinement the observations were arbitrarily categorized into one of eleven themes: DNA and epigenetics, Mitochondria, Nucleus, Immune system, Protein and metabolism, Lysosome and degradation, Cell cycle, Cytoskeleton, Extracellular Matrix (ECM), Cell mechanics, and Cell signaling. The following search term was used in Web of Science and PubMed for which each aging sign was inserted as the subject:

*(subject) AND ((Gravity) OR (hypergravity) OR (microgravity) OR (micro‐gravity) OR (hypergravity) OR (unloading) OR (mechanical loading) OR (spaceflight)) NOT (radiation)*

The search was performed in the period between October 2022 and March 2023. Radiation was excluded in order to obtain more refined results with a focus on altered gravity instead of radiation. Of course, any research performed in spaceflight could be affected as well by radiation or by the combination of radiation and altered gravity. Furthermore, broad genetic screening studies were outside the scope of this literature review.

### 2.2 Aging Sign Optimalization

First, the items for aging were optimized, decreasing the number of aging signs from 209 to 165. Consequently, the total number of articles to be analyzed was reduced from 19,885 to 12,980. Hence, for this study, 12,980 articles were subsequently evaluated. The majority of the aging signs that got discarded discuss signs in larger systems like the elasticity of the aorta or the shear modulus of an entire muscle. Furthermore, upon closer inspection of all the signs, multiple aging signs described the same phenomenon in a different setting or wording. For example, red blood cell (RBC) rigidity and RBC deformability were combined, and actin thickness and cytoskeleton thickness were merged into a single aging sign as well. Finally, a few signs were removed as the articles describing the phenomena used either insufficient controls or little scientific evidence was provided to substantiate the claims.

### 2.3 Results Criteria

The remaining 165 items were evaluated in‐depth for gravity‐related research irrespective of cell type and in vivo or in vitro setup. Many articles were discarded as they were irrelevant and not related to gravity research. Especially, the term “mechanical loading” and, to some extent, also the term “unloading” caused many unrelated articles in the search results. Some gravity‐related articles could also be debated. For example, hindlimb unloading, a method designed to examine bone and muscle: we do include some references to this technique of microgravity simulation; however, some organ systems and types of tissue need more research to substantiate this simulation paradigm as fully validated [43]. Likewise, we also excluded articles using magnetic levitation to simulate microgravity and articles that simulate hypergravity by compression. Furthermore, articles with plants, bacteria, viruses, or fungi as subjects were discarded, as all the previously determined aging signs described in the five reference articles exclusively reviewed the aging of humans and animals and their respective tissues and cells.

### 2.4 Searching Approach

For the in‐depth investigation, both PubMed and Web of Science are used to search the aging sign with the search term. The item is searched in different spellings or words and with full names and abbreviations (for example, heat shock transcription Factor, HSF‐1, HSF1). For each aging sign, a maximum of five gravity‐related articles were selected to keep the size of this research manageable. As previously mentioned, the terms “mechanical loading” and “unloading” induced many unrelated articles in the search results. If more than 50 results per search engine were retrieved for a specific item, the number of results was reduced by eliminating the terms “mechanical loading” and “unloading” from the search term. If less than five relevant articles were found with the remaining search term, the terms were added once again to the search term, and all found articles were scanned for relevance. When more than five relevant articles were found in the search results, a decision tree was applied to guide selection. Both the decision tree and the rationale behind the selection criteria are presented in Figure S1 of the Supporting Information.

### 2.5 Scoring

**FIGURE 1:**
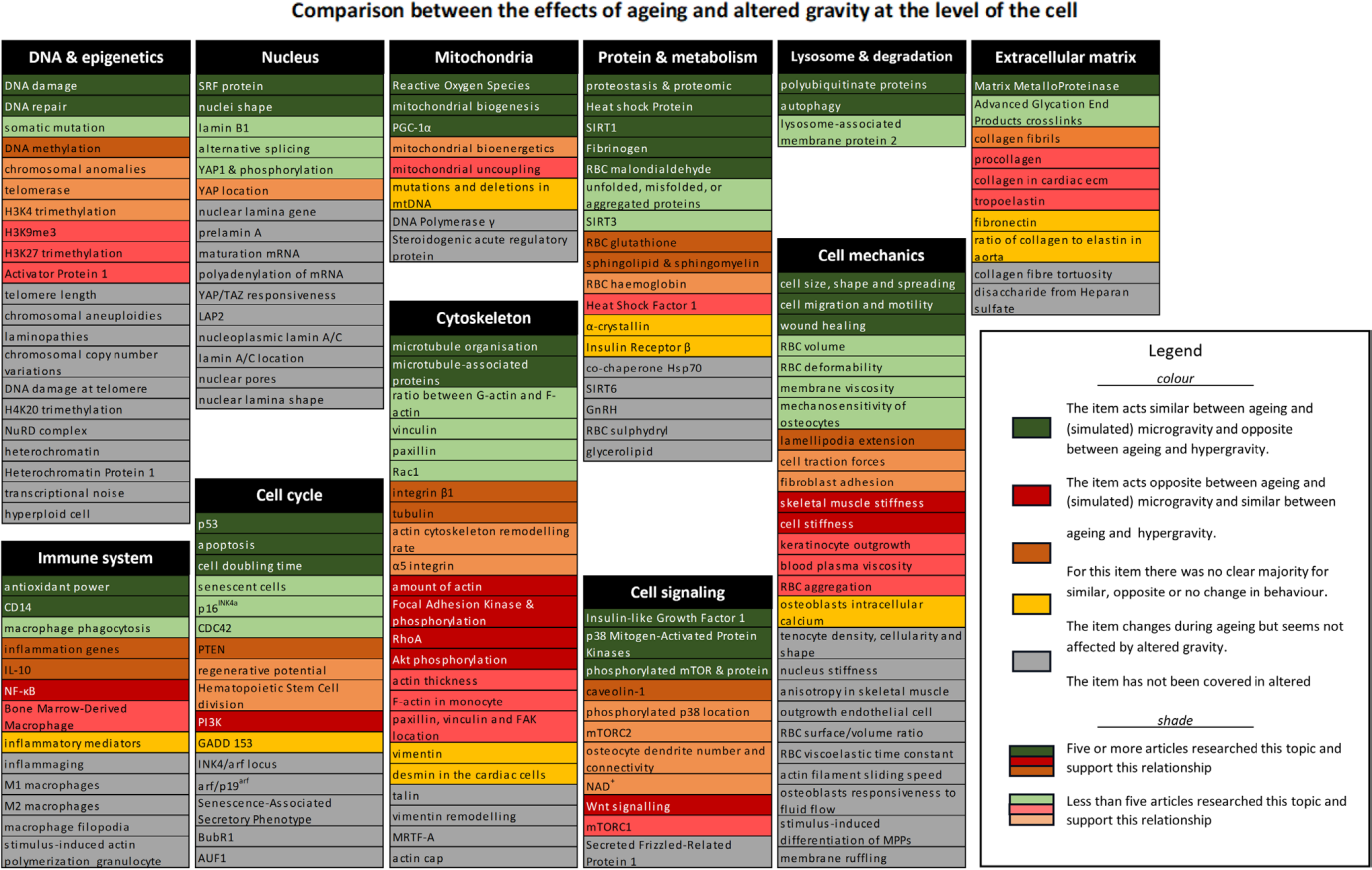
A summary of comparisons between the effect of biological aging and altered gravity for the 165 identified signs of aging at a cellular level. Each theme is presented in black, followed by the items associated with that theme. The colors describe similarities and dissimilarities between the effect of altered gravity and aging by topic, while the shade indicates how many articles support this relationship.

For each item, the number of gravity‐related articles was reported until five. If there were more than five relevant articles, “5+” was noted, and five articles were selected using the decision tree. For each aging sign, it was noted how it changes with altered gravity. Color coding was used to describe similarities and dissimilarities between the effects of altered gravity and aging. If the item behaved similarly in (simulated) microgravity and aging and opposite with hypergravity a green color was assigned to the item. If the item acted similarly in hypergravity and aging and opposite in (simulated) microgravity a red color was given. Yellow was assigned to items that do not seem to change with altered gravity. Furthermore, if no gravity‐related articles were found for that item, gray was given. The color depends on the majority of the outcome for the found results. However, if there was no clear majority in literature, the color orange was given. Besides a color, a shade was given to indicate the number of articles. Items that had less than five articles were given a lighter shade than articles that had more than five articles. The final results are plotted in Figure 1.

## 3 Results

### 3.1 Comparing Aging and Altered Gravity

An overview of the study results is provided in Figure 1. The inner circle displays the themes, and the outer circle shows all items belonging to these themes. The colors describe similarities and dissimilarities between the effect of altered gravity and aging per item, while the shade indicates how many articles support this relationship. The following sections detail the key findings for each theme, while all observations and accompanying references are available in Table S1 of the Supporting Information.

### 3.2 DNA and Epigenetics

For the theme DNA and epigenetics, 634 references were screened. Many of the 21 items of this theme, like transcriptional noise, telomere DNA damage, or laminopathies, still require investigation in altered gravity, while other items, like somatic mutations or the trimethylation at histone H3, are supported by only a few articles. DNA damage increases with aging and in simulated microgravity for in vitro studies with human and rodent cells [42, 44, 45, 46, 47, 48, 49, 50, 51]. Deficiencies in the DNA repair mechanisms increase in human and mouse cells in (simulated) microgravity and may accelerate aging [47, 48, 52, 53, 54, 55, 56, 57].

DNA methylation decreases with age, but no clear majority in the effect is found for altered gravity [58]. In astronauts no significant change in DNA methylation was found, and for human cells in simulated microgravity there appears to be either an increase or a decrease, depending on the region of DNA investigated [59, 60, 61]. In rodent cells in real microgravity, the total DNA methylation seems to increase, while no change was observed in simulated microgravity [62, 63].

### 3.3 The Nucleus

The Nucleus is the theme with the relatively least investigated items. Although 686 references were analyzed for this theme, only six of the 16 items have been explored in altered gravity conditions. Serum Response Factor (SRF) promotes muscle regeneration and is found to reduce with age [44]. For studies with humans and rats in vivo and in vitro in (simulated) microgravity, SRF appears to be downregulated [64, 65, 66]. With age, more nuclei have abnormal shapes, such as folds, stretching, blebbing and fragmentation [67, 68]. In real and simulated microgravity, more nuclei with folds, blebbing and fragmentation were also observed in experiments with human, rodent, and porcine cells [51, 69, 70, 71, 72]. Of the 16 signs in the Nucleus theme, there were no opposite responses found, and only one, the location of the transcriptional coregulator YAP, displayed an ambiguous response in simulated microgravity compared to aging.

### 3.4 Mitochondria

A relatively large number of items in the theme Mitochondria behave similarly with aging and in (simulated) microgravity and have been investigated in more than five articles. For this theme, 669 articles were investigated. Research in mitochondrial bioenergetics in altered gravity is inconclusive, with some observations suggesting an increase in bioenergetics in human oligodendrocytes and others a decrease in human osteoblasts in simulated microgravity [73, 74]. However, another indicator of mitochondrial effectiveness, mitochondrial biogenesis, does seem to show a majority. Decreased mitochondrial biogenesis is observed in the rat soleus or gastrocnemius muscles for simulated microgravity and with increasing age in the human vastus lateralis [75, 76, 77, 78, 79]. Overexpression of PGC‐1α, a regulator of energy metabolism, appears to extend longevity in *Drosophila* [80]. Hindlimb unloading with rodent and bedrest studies with humans indicated a decrease in PGC‐1α in the gastrocnemius, soleus, and vastus lateralis [77, 78, 81, 82, 83]. Reactive oxygen species (ROS) increases in both aging and simulated microgravity for human and rodent cell lines [1, 84, 85, 86, 87, 88].

### 3.5 Immune System

A total of 1204 references were reviewed for the immune system theme. It contains quite some items that are yet to be investigated in altered gravity, especially items regarding macrophages. With age, overexpression of inflammation genes seems to increase [89, 90]. Different results appear for varying methods in altered gravity. Simulated microgravity in vitro studies show upregulation of inflammation‐related genes [35, 91, 92]. However, in a real microgravity in vitro study, no changes were observed [35]. In contrast, an in vivo experiment in microgravity showed downregulation, as did an in vitro study under hypergravity conditions [93, 94].

The activation of nuclear factor kappa‐light‐chain‐enhancer of activated B cells (NF‐kB), a transcription factor playing a crucial role in the cellular response to inflammation, seems to increase with age, and inhibition of this pathway appears to extend longevity and delay some symptoms of progeria in mice [95, 96, 97]. Results for in vitro studies in microgravity and simulated microgravity suggest opposite behavior, as NF‐kB‐related genes are downregulated and have decreased activation [31, 36, 64, 98, 99]. Although one of the real microgravity studies with human breast cancer cell lines indicated an upregulation of NFKB1, NFKB3, NFKBIA, and NFKBIB but a reduced protein content [36].

Surprisingly, while increased inflammation is often related to the negative effects seen in microgravity, the majority of inflammatory mediators like IL‐1β, IL‐17, and IL‐4 do not change significantly in in vivo studies of humans or mice during spaceflight [100, 101]. Similarly, in simulated microgravity for both in vitro and in vivo studies, many mediators seem unchanged, while it appears to rise with aging [102, 103, 104, 105]. For a few inflammation mediators, like interleukin 10 (IL‐10), results appear ambiguous. In whole blood from astronauts, IL‐10 appears to remain unchanged, increase, or decrease, depending on the study [100, 106, 107]. Simulated microgravity resulted in an upregulation in IL‐10 in the murine macrophage cell line, and hypergravity with whole blood samples from mice led to the downregulation of IL‐10 [108, 109]. Antioxidant power seems to diminish with age, and for most articles in simulated and real microgravity for in vivo and in vitro studies with human or murine subjects [88, 110, 111, 112, 113]. The expression of the phagocytosis receptor CD14 is decreased in bone marrow‐derived macrophages of aged mice [114]. Likewise, CD14 expression decreases in the blood of astronauts and in primary human macrophages exposed to real microgravity [115, 116, 117]. Under simulated microgravity conditions, a decrease in CD14 was found in gingival tissue of rhesus macaques, while no changes were observed in an in vitro experiment with human limbal fibroblasts [118, 119].

### 3.6 Protein and Metabolism

The theme Protein and Metabolism has the most items that behave similarly in (simulated) microgravity and with increasing age, which are also supported by a considerable number of articles. In total, 1227 references were analyzed for this theme. Studies have investigated protein expression under conditions of altered gravity, including real and simulated microgravity as well as hypergravity. Just as with aging, they all seem to indicate altered proteomics for both in vivo and in vitro experiments with humans, rodents, or flies [37, 110, 120, 121, 122, 123, 124]. Overexpressing heat shock proteins (HSPs) seems to increase longevity [125, 126]. In simulated and real microgravity, HSPs seem to have reduced mRNA expression in human and rat cell lines as well as in in vivo rat studies, while in hypergravity, it seems to increase in the rat soleus [27, 127, 128, 129]. Another study with the lenses of embryo zebrafish also detected an increase in HSPs for hypergravity but found an increase in simulated microgravity conditions as well [130].

Moderate upregulation of Sirtuin 1 (SIRT1), an enzyme located mainly in the nucleus that deacetylates transcription factors contributing to cellular regulation, appears to improve healthy aging [131, 132]. Although some articles contradicted each other, the majority of the studies investigating SIRT1 mention reduced expression of mRNA and protein in the gastrocnemius muscle of rodents and in rat mesenchymal stem cells (MSC) in simulated microgravity [77, 133, 134]. An increase of SIRT1 mRNA and protein was found in the gastrocnemius muscle of mice during hindlimb unloading and in *C. elegans* in real microgravity [135, 136].

The coagulation factor fibrinogen in human blood plasma and its synthesis rate seem to increase in microgravity, bedrest studies, and with increasing age [38, 137, 138, 139, 140]. Short dry immersion did not seem to alter fibrinogen levels in human blood plasma [38]. Another bedrest study reported no change in fibrinogen levels among men, whereas an increase was observed in women [139]. Regarding fibrinogen levels in the blood plasma of astronauts, mission duration seems to be influential. While short flight or early flight datapoints indicate increased fibrinogen, decreased fibrinogen was detected after six days [38, 138, 141].

Sphingolipids tend to increase with age, while glutathione decreases with age [142, 143]. However, although both items have been discussed in multiple articles in simulated and real microgravity, the behavior of plasma membrane lipid bilayer‐associated sphingolipids and red blood cell glutathione in both in vitro and in vivo altered gravity studies remains inconclusive. Two articles on sphingolipids in hypergravity and real microgravity in rodents found no change, while two others in microgravity reported varying levels in rodent blood or liver [40, 144, 145, 146]. Simulated microgravity showed an increase in sphingolipids in rat liver and a decrease in human gastric cancer cells [40, 147]. Glutathione levels for in vitro studies with human RBC tend to decrease, and the redox glutathione capacity is altered [88, 148, 149]. No change was found in two other articles investigating glutathione levels in human blood in simulated and real microgravity [150, 151].

### 3.7 Lysosome and Degradation

Although this theme only contains three items, they all seem to behave similarly with aging and in (simulated) microgravity, for which 220 articles were screened. Polyubiquitinated proteins and mRNA seem to accumulate with age in mice muscle cells and in both real and simulated microgravity in the gastrocnemius or soleus muscles of rodents [28, 152, 153, 154, 155, 156]. Autophagy activation appears to increase lifespan [157, 158, 159]. Multiple in vitro studies with either murine or human cell lines indicate increased autophagy in real and simulated microgravity [160, 161, 162]. For in vivo studies in microgravity, autophagy related genes were upregulated in mice livers but downregulated in mice quadriceps [163, 164].

### 3.8 Cell Cycle

For the theme Cell Cycle, 1801 references were analyzed, making it the theme with the most search hits per item. Many items in this theme are closely related, yet do not seem to always have similar behavior in altered gravity and with aging. Inactivation of phosphoinositide 3‐kinase (PI3K), a key effector in signal transmission, would increase longevity [165]. In rodent pre‐osteoblasts and murine muscle and bone, PI3K activation is inhibited under simulated microgravity conditions [166, 167, 168]. In contrast, PI3K activation is more pronounced in rodent pre‐osteoblasts under hypergravity conditions [166]. For in vitro studies involving human choroidal vascular endothelial cells or leukocytes under simulated microgravity, PI3K activation either increases or shows no change [169, 170].

Numerous experiments in both simulated and real microgravity have been conducted involving tumor protein P53 (p53). P53 levels appear to increase with age and promote cell cycle arrest to prevent the propagation of damaged cells [54, 171, 172, 173]. In vitro studies involving rodent and human cell lines for simulated and real microgravity report an increase of p53 [31, 174, 175]. In vivo experiments with either rat liver or mouse thymus in microgravity found, respectively, a decrease or an increase in p53 [176, 177].

Apoptosis has been found to increase with advancing age and is frequently investigated in real microgravity [114, 178]. In both simulated and real microgravity studies, apoptosis or apoptotic genes were reported to be upregulated in mouse eyes or lungs and in human cell lines like Jurkat cells [122, 179, 180, 181, 182]. Cell doubling time increases with age, seems to decrease in hypergravity, and shows varying results in simulated microgravity depending on cell type [183]. Human cells, including hMSC and umbilical vein endothelial (HUVEC), were found to have a reduced doubling time in simulated microgravity [184, 185]. In contrast, murine cells like rat bone marrow‐derived MSC reported an increased doubling time in simulated microgravity [186, 187]. Hypergravity experiments with the rodent BMSCs and C2C12 myoblasts showed a decreased doubling time [186, 188].

The overexpression of the tumor suppressor phosphatase and tensin homolog (PTEN) seems to increase longevity, while results for PTEN in altered gravity are quite divergent [189]. In simulated microgravity, PTEN levels in human cancer cell lines were either increased or unchanged, whereas in human primary cells such as peripheral blood lymphocytes, PTEN levels decreased [190, 191, 192, 193, 194].

### 3.9 Cytoskeleton

The theme Cytoskeleton seems to have the most items that act opposite between aging and (simulated) microgravity, which are also supported by more than five articles. In total, 1837 articles were screened for this theme. In multiple cell types, including HUVECs and mouse embryonic fibroblasts, as well as in the soleus and biceps brachii of rats, actin filaments or their mRNA expression levels were found to decrease in both real and simulated microgravity and increase in hypergravity [65, 195, 196, 197, 198]. In contrast, actin appears to increase with aging for in vitro studies with vascular smooth muscle cells of monkeys, human dermal fibroblasts and endothelial cells, and in in vivo studies with human vastus lateralis and the soleus of rodents [199, 200, 201, 202].

A key protein involved in focal adhesion signaling is focal adhesion kinase (FAK) and its activated form, phosphorylated FAK (p‐FAK). Real and simulated microgravity seem to reduce p‐FAK in the soleus of astronauts, hMSCs, and a mouse pre‐osteoblasts cell line [203, 204, 205]. FAK either remains unchanged in hMSCs or decreases in the astronaut soleus in microgravity [203, 204]. In hypergravity conditions, FAK and p‐FAK increase in a time‐ and gravity‐dependent manner for primary human tendon cells or for the cell line hMEC‐1 [206, 207]. This mirrors the observed increase in FAK and p‐FAK levels with age in human fibroblasts and in the aorta wall of rats [208, 209].

Ras homolog family member A (RhoA) is a member of the Rho family GTPases, which regulate actin crosslinking and stress fiber formation that are critical for cell shape, motility, and division [57]. It appears to increase in the skeletal muscle of aged mice, and it seems also to increase in adult human cardiovascular progenitor cells in microgravity but decrease in neonatal cells in similar conditions [57, 210]. In simulated microgravity, RhoA or its activity is decreased in human and mouse cell lines, whereas RhoA activity is increased in bovine endothelial cells in hypergravity [196, 211, 212, 213].

Another item that behaves differently in aging and altered gravity is phosphorylated protein kinase B (p‐Akt). Phosphorylation of Akt promotes protein synthesis, muscle hypertrophy, and cell survival and counteracts the loss of muscle protein [214]. Activation of Akt was found to increase in the soleus of aged mice [214]. An increase was also reported in the masseter of rats in microgravity, while a decrease was found in the tibialis anterior [215]. In hypergravity, p‐Akt in the tibialis increased but remained unchanged in the soleus [216]. Under simulated microgravity conditions, activated Akt seems to decrease in the vastus lateralis of human subjects and in a mouse myoblast cell line, yet shows no change in human cardiovascular progenitor cells [217, 218, 219].

During aging, a larger percentage of microtubule show an abnormal organization and have a higher catastrophe rate [220, 221]. Furthermore, the fractions of microtubule‐associated proteins increase [222]. Experiments conducted in real or simulated microgravity report a lack of self‐organization, poor organizing centres, and an altered or disrupted microtubule network in in vitro studies using primary cow brain cells, mouse osteoblasts, and human and insect cell lines [29, 69, 180, 223, 224]. Additionally, microtubule‐associated proteins seem to increase in simulated microgravity research with human or rat MSC cells and a mouse pre‐osteoblast cell line [225, 226, 227]. Hypergravity conditions appear to increase these proteins in the inner ear of mice [228]. However, some studies found no change in microtubule‐associated proteins in the soleus of mice in simulated microgravity, nor in organization under hypergravity conditions [29, 229]. Although tubulin appears well investigated in real microgravity, results are contradictory. Both α‐tubulin and β‐tubulin expression were found to be downregulated in human and rodent cells, mirroring senescent human fibroblasts [230, 231, 232]. However, an increase in α‐tubulin or no effect under microgravity was also reported for a human macrophage cell line and human fibroblasts, along with a decrease in tubulin mRNA levels in human endothelial cells in hypergravity conditions [179, 233, 234].

Finally, a decrease in integrin β1 is reported in senescent human fibroblasts and aged human skin, while results for altered gravity are inconclusive and seem to vary between species [208, 235]. Experiments conducted in microgravity and hypergravity with rat osteoblasts do not report a change in integrin β1, while mRNA and total protein levels increase in human chondrocytes under microgravity conditions [231, 236, 237]. In simulated microgravity experiments, it either increases in human dermal fibroblasts or decreases in HUVECs and a human breast cancer cell line [238, 239].

### 3.10 Extracellular Matrix

Most aging signs in the theme ECM are either not investigated or are supported by only a few papers, while 1103 references appeared as search hits for this theme. Fragmentation of collagen fibrils is observed with advanced age in human fibroblasts and skin [235, 240, 241]. The literature shows conflicting findings on how altered gravity impacts collagen fibrils. Some studies report no significant changes in the aortic wall and tibial growth plates of rodents exposed to both real and simulated microgravity [32, 242]. In contrast, other research findings indicate a decrease in fibril size or increased fragmentation in the tendon or chondrocytes of mice and in the soleus of astronauts subjected to hypergravity or microgravity conditions [243, 244, 245].

Matrix metalloproteinases (MMP) break down ECM for remodeling [246]. With age, there is an increase in MMPs [235, 241, 247]. Similarly, increased levels of MMP‐1, MMP‐2, MMP‐3, MMP‐8, MMP‐9, and MMP‐10 were found in human fibroblasts, a human endothelial cell line, human saliva, and blood, as well as in the pelvic bone of mice under (simulated) microgravity and hypergravity conditions [207, 238, 248, 249]. Fibronectin, a cell surface adhesion protein, facilitates the binding of various cell types to other ECM components and was found to increase with age [250, 251]. However, most articles report no significant changes in fibronectin in quadriceps and pre‐osteoblasts of mice and in human fibroblasts subjected to microgravity or hypergravity [164, 234, 250]. One study reported an increase in fibronectin levels in rat osteoblasts in microgravity after 4 days but no change after 5 days [231].

### 3.11 Cell Mechanics

Interestingly, the largest theme with the most signs for aging is also one of the themes with the most ill‐investigated items for gravity‐related research. In total, 2439 articles were screened for the theme Cell Mechanics. The stiffness of skeletal muscle fibers in rodents seems to increase with age [252, 253]. Findings for altered gravity vary between simulated and real microgravity and for different muscle groups. The stiffness of the soleus in rhesus monkeys and rodents appears to increase for hypergravity and decrease for simulated microgravity and no changes were reported in real microgravity [254, 255, 256, 257]. The tibialis anterior in rodents seems to increase in stiffness for both hyper‐ and microgravity [254, 257]. In contrast, the stiffness of the medial gastrocnemius and its sarcolemma in rhesus monkeys and rodents was reported to decrease in simulated and real microgravity [255, 256]. Cell stiffness in numerous other cell types ranging from human epithelial cells to fibroblasts and chondrocytes also appears to increase with aging [258, 259, 260, 261, 262, 263]. Just as with the stiffness of skeletal muscle fibers, only a slight majority of studies show opposite behavior between aging and (simulated) microgravity and similar behavior between aging and hypergravity. The variation observed in the results seems to depend on the type of cell and species studied in the experiment. The cell stiffness of HUVECs and human osteoblast was found to be reduced in simulated gravity, whereas it increased for rat MSCs in simulated microgravity [264, 265, 266]. Additionally, the stiffness of cardiomyocytes in rodents remains unchanged in real microgravity but decreases in hypergravity [254, 257].

The motility of human and rodent fibroblasts appears to reduce with aging [183, 267, 268, 269]. A similar trend is observed under simulated microgravity conditions in a human monocyte cell line, a mouse fibroblast cell line, leech skin, and primate vascular smooth muscle cells. These studies report a decrease in migration distance, migration velocity, and number of cells migrating [270, 271, 272, 273]. However, hypergravity conditions result in increased directed motion, as well as increased total migration distance in low serum media [273]. Additionally, the motility of human embryonic lung cells subjected to real microgravity remains unchanged [274]. Wound healing seems to delay with increasing age in humans [275, 276]. Similarly, under altered gravity, most articles also indicated impaired wound healing. Human and rodent fibroblasts subjected to simulated microgravity and hypergravity showed delayed wound closure in scratch assays [271, 272, 277]. Experiments conducted in real microgravity have primarily focused on larger physiological systems rather than the cellular perspective. Nevertheless, studies have observed impaired wound healing in the granulation tissue of rodents, while no significant changes were noted during wound healing in the crushed gastrocnemius of rats [278, 279].

Fibroblasts from aged human skin or mouse tendon spread less, become rounder in shape and reduce in size [183, 240, 241, 280]. A similar pattern is observed in altered gravity, where the cell area decreases in a human monocyte cell line and mouse fibroblasts subjected to (simulated) microgravity, while in hypergravity human fibroblasts appear to spread more [197, 230, 281]. Conversely, endothelial cells increase in size with age, as well as in microgravity [282, 283]. Additionally, another study reported an increase in cell area in human osteoblasts subjected to real microgravity [284].

Lamellipodia extension seems to decrease with age in human skin fibroblasts [269]. The literature on altered gravity primarily focuses on in vitro studies with human (cancer) cell lines yet reports conflicting findings on how altered gravity impacts lamellipodia. An increase in lamellipodia formations, and occasionally filopodia or pseudopodia, was observed in endothelial cells and breast cancer cells in simulated and real microgravity, as well as in neuroblastoma cells in hypergravity [29, 285, 286]. After 31 parabolas of parabolic flight, which include phases of microgravity and hypergravity, an increase in lamellipodia was observed in prostate cancer cells [287]. Additionally, neuroblastoma cells remain unchanged after simulated microgravity, whereas monocytes show a decreased level of pseudopodia under real microgravity conditions [29, 230].

### 3.12 Cell Signaling

A total of 1171 references were reviewed for the Cell Signaling theme. Compared to the other themes, Cell Signaling contains many items that have been examined in more than five papers. Insulin‐like growth factor 1 (IGF‐1) is a hormone that primarily mediates growth hormone (GH)‐stimulated somatic growth and also facilitates GH‐independent anabolic responses in various cells and tissues [288]. IGF‐1 levels tend to decrease in various tissues of aged mice [289]. Likewise, during both real and simulated microgravity, these levels appear to reduce in rodent osteoblasts, blood plasma, and the soleus muscle but increase in the extensor digitorum longus (EDL) muscle [290, 291, 292, 293]. An increase in IGF‐1 was also reported in the tibia of rats under real and simulated microgravity [30].

The p38 mitogen‐activated protein kinase (MAPK) functions as a crucial signal transduction mediator involved in regulating inflammation, the cell cycle, cell death, development, cell differentiation, senescence, and tumorigenesis across various cell types [294]. Activation of p38 MAPK increases with age in human granulosa cells, skeletal muscle, and macrophages as well as in the aorta of rodents [209, 295, 296, 297]. A rise in activation was observed in human MSCs, rodent macrophages, and neural crest stem cells experiencing (simulated) migrogravity [204, 298, 299]. A decrease in activation was reported in a human promonocytic cell line infected with enteropathogenic *E. coli* bacteria under simulated microgravity [300]. Additionally, no change in p38 MAPK activation was detected in monocytes extracted from human blood [301].

While little research on Mammalian Target of Rapamycin complex 1 (mTORC1) and mTORC2 is performed in altered gravity, more papers investigate phosphorylated mTOR (p‐mTOR) and total mTOR protein. mTOR signaling is essential for controlling translation, lipid synthesis, nucleotide synthesis, lysosome biogenesis, nutrient sensing, and growth factor signaling [302]. In simulated microgravity and with advanced age, p‐mTOR seems to reduce in the soleus of rats [214, 303]. A decrease in p‐mTOR and mTOR protein is observed in the human vastus lateralis and rodent pre‐osteoblasts under simulated microgravity conditions [227, 304]. Interestingly, studies that investigated the gastrocnemius muscle of rats subjected to hindlimb unloading reported an increase in p‐mtor [305, 306].

The canonical Wnt pathway, which is involved in regulating cell proliferation, survival, and cell fate, shows increased activation with aging in mouse muscle stem cells [307, 308]. For real and simulated microgravity, it seems that the signaling pathway is suppressed in goldfish scales, as well as in rodent pre‐osteoblasts and bone marrow‐derived MSC [33, 205, 309]. Another study in simulated microgravity reported altered Wnt signaling for various components of the pathway [310]. In contrast, human cardiovascular progenitor cells cultured on board of the International Space Station (ISS) showed promotion of the Wnt pathway in adult cells and suppression in neonatal cells [57]. Caveolin‐1 (CAV1) is an intracellular signaling protein involved in various processes, including signal transduction, lipid transport, and metabolic regulation [311]. Expression of CAV1 increases for senescent cells in human diploid fibroblasts [208]. Results on CAV1 under altered gravity conditions seem divided and appear to be dependent on the duration of the experiment for research conducted under real microgravity conditions. No significant change was observed for CAV1 in a human thyroid cancer cell line after several minutes during sounding rocket flight, while and increase in levels was found during the hypergravity phase of the flight [312]. CAV1 levels in human chondrocytes were increased after 31 parabolas in parabolic flight, while no change was observed in the hypergravity phase of the flight [237]. Similarly, after 91 days in real microgravity, levels of CAV1 were increased in the thyroids of mice [313]. Studies with HUVECs under simulated microgravity conditions show either an increase or decrease of CAV1 [314, 315].

### 3.13 Overall Effect

**FIGURE 2:**
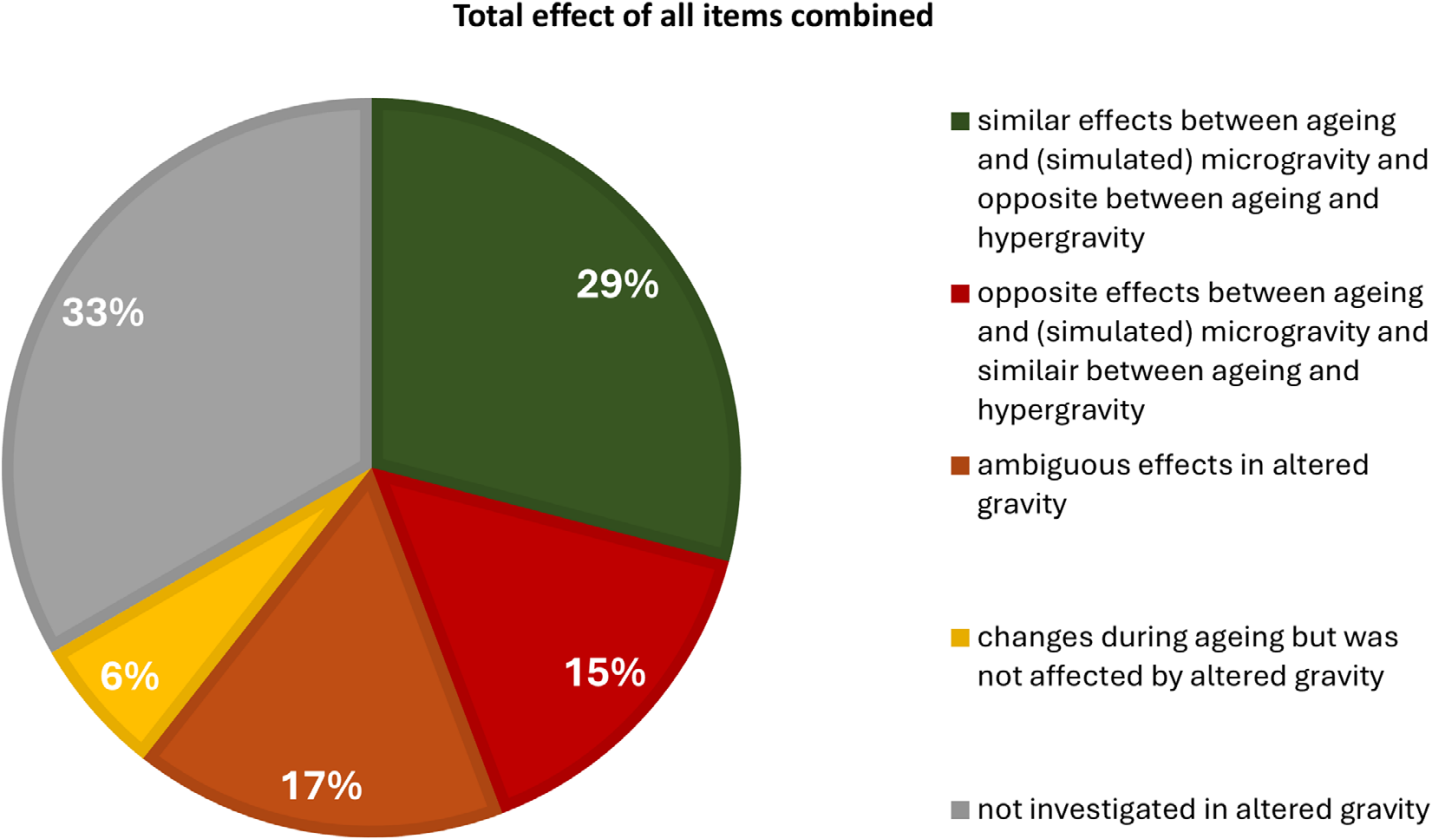
Summary for all 165 signs of aging at the level of the cell, taken from Figure 1, showing the overall relationship between the effects of biological aging and altered gravity.

Figure 2 summarizes the overall result for the 12,980 examined articles. A significant group of items relevant to biological aging are still to be explored in altered gravity conditions. Nevertheless, in general, it seems that 29% of the papers on aging and (simulated) microgravity have similarities at a cellular level. Surprisingly, a significant portion, approximately 32%, exhibited opposite and more ambiguous effects.

### 3.14 Utilized Methods

Several methods are used to investigate samples under altered gravity conditions. For simulated microgravity, the most commonly applied methods include clinorotation [24], the random positioning machine, (head‐down tilt) bed rest [316] or dry immersion [317] studies, and hindlimb unloading (mostly for the musculoskeletal system) [318]. Simulated microgravity is also the predominant approach in gravity‐related research, as 56% of the articles used to generate the data for Figure 1 employed this method. Interestingly, despite the high costs associated with real microgravity experiments such as orbital spaceflight, sounding rockets, and parabolic flights, a greater proportion of studies was conducted under real microgravity conditions (30%) compared to hypergravity (14%).

## 4 Discussion

**FIGURE 3:**
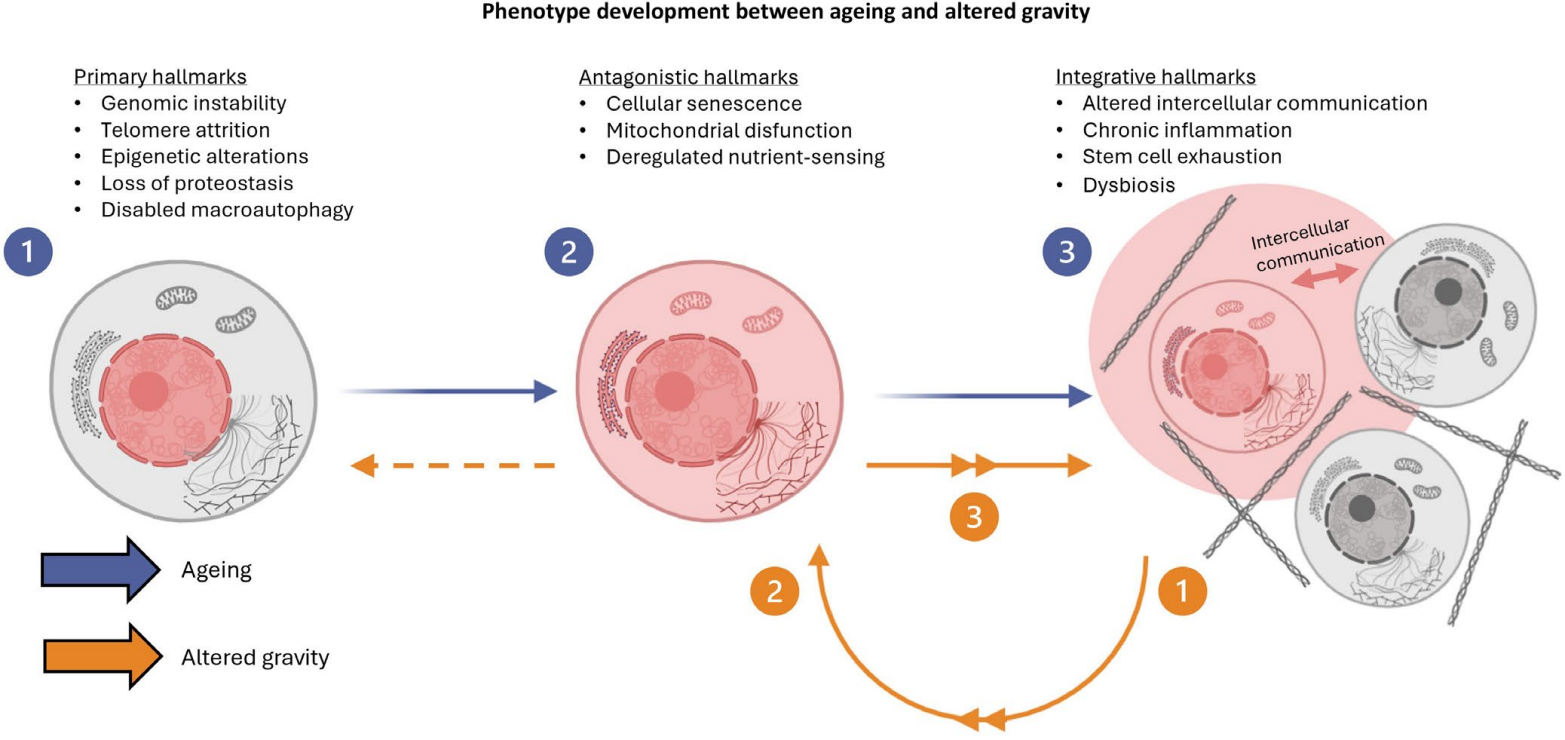
Proposed framework of the phenotype development in altered gravity compared to biological aging mechanisms. In blue: The aging mechanism as described by López‐Otín C. et al. (1) Aging starts as damage (shown in red) in primary hallmarks located mainly in the nucleus. (2) Antagonistic hallmarks act as countermeasures but will eventually become detrimental themselves, resulting in damage throughout the cell. (3) Integrative hallmarks result from the Primary and Antagonistic hallmarks and propagate damage to the cell's enviroment. In orange: Our proposed framework of the phenotype development in altered gravity. (1) Altered gravity results in poor signal translation of the mechanical environment, i.e., altered intercellular communication. (2) Antagonistic hallmarks respond to the altered intercellular communication as they act as countermeasures but will eventually become detrimental themselves. Simultaneously, this altered environment also affects the primary hallmarks. (3) Antagonistic hallmarks and, to some extent, Primary hallmarks sustain Integrative hallmarks further. We propose that unlike in biological aging, where Primary hallmarks often initiate the process, the spaceflight environment may trigger a feedback loop between Antagonistic and Integrative hallmarks, which in turn contributes to the accelerated aging phenotype. Upon returning to 1 g conditions, these effects are reversed, as the restoration of proper signal transduction terminates the cycle.

Biological aging and (simulated) microgravity seem to have similar phenotypes at higher‐system/organ level and to some extent also at a cellular level. We did find, however, a relatively large number of opposite responses as well. One of the main differences between biological aging and microgravity is reversibility. While astronauts experience a lot of aging phenotypes, after a considerable recovery period, most effects will eventually be reversed and return to pre‐flight values. For aging, this is not the case. Lopez‐Otin et al. propose in their latest article 12 hallmarks of aging [319]. This classification remains arbitrary as the distinction between hallmarks is intrinsically diffuse and interdependent [319]. Although the hallmarks are strongly related to one another and form a complex entanglement, a certain pattern is recognized that divides the hallmarks into Primary hallmarks, Antagonistic hallmarks, and Integrative hallmarks. López‐Otín et al. describe how biological aging originates as damage in Primary hallmarks. Initially, the Antagonistic hallmarks act as countermeasures to this damage, but when these responses become chronic or exacerbated, they become detrimental themselves. Finally, Integrative hallmarks are the outcome of the Primary and Antagonistic hallmarks and are responsible for the functional aging phenotype. We speculate that altered gravity changes the signal translation of the mechanical environment. If altered gravity causes altered intercellular communication, this could trigger the Antagonistic hallmarks. This, in turn, may establish a self‐reinforcing feedback loop between the Antagonistic and Integrative hallmarks. Although this loop can also influence the Primary hallmarks, it is not primarily driven by them, as in aging. Therefore, upon returning to Earth's gravity, this loop will not persist, allowing proper signal transduction to be restored (Figure 3).

Key players in cellular mechanotransduction are the ECM, focal adhesions, the cytoskeleton, the linker of the nucleoskeleton and cytoskeleton complexes (LINC), and the nuclear lamina. The nuclear lamina is a network inside the nucleus close to the nuclear membrane consisting of lamins and lamin‐associated proteins and connects the chromatin to the nuclear envelope [320]. Defects and mutations in the nuclear lamina and the genes encoding for it seem to result in accelerated aging [321, 322, 323, 324]. These mutations cause laminopathies, such as progeria and Hutchinson‐Gilford progeria syndrome (HGPS) [325]. Restoring the nuclear lamina in HGPS patients can increase their lifespan [326]. Defects in the precursor of one of the main components of the nuclear lamina, Lamin A/C, can also lead to accelerated aging, and the precursor's mutant form, progerin, appears to accumulate with age [327, 328]. Lamin A/C is part of the link enabling mechanotransduction from the extracellular environment through the focal adhesions, the cytoskeleton, and the LINC complexes into the nucleus [18]. Force transmission is impaired in cells with mutations in the LMNA gene [329]. Unfortunately, not a single article was found in altered gravity investigating possible defects in the nuclear lamina or mutations in the gene. Another property of Lamin A/C that changes with age is its location in the nucleus. During aging, the location of Lamin A/C will shift from throughout the nucleoplasm to the nuclear rim [330, 331]. Koaykul et al. observed altered Lamin A/C polarization in simulated microgravity and hypothesize that this could result in altered histone modification [226]. Lamins A/C also contribute to the mechanical stiffness of the nucleus and the cell [332, 333]. Investigating Lamin A/C in altered gravity could provide more insight about its mechanotransduction role but potentially also shed more light on the numerous articles of cell stiffness in altered gravity for which the results remain inconclusive.

The LINC complex is also an important component mediating mechanotransduction by connecting the nuclear lamina with the actin cytoskeleton, intermediate filaments, and microtubules. In HGPS patients disrupting LINC complex reduces the effect of accumulating progerin [334]. Defects in the genes encoding for LINC complex proteins results in a similar phenotype seen in laminopathies [335]. Little research has been performed on LINC complex involvement with aging and even less is achieved with altered gravity [336]. Neelam et al. observed increased nuclear height and differential gene expression in simulated microgravity in cells with disrupted LINC complexes [70]. The increase in nuclear height may be caused by a disrupted actin cap. The LINC complex is also connected to the actin cap which consists of perinuclear actin fibers wrapping around apical surface of the nucleus and form upon inducing low mechanical stresses [337]. Cells depleted of the nuclear envelope associated protein nesprin were unable to form actin caps upon shear stress formation [337]. For progeroid mice the actin cap appears disorganized or is completely eliminated [337]. Surprisingly, no results for the actin cap in altered gravity were found, while it plays a critical role in mechanosensation and mechanotransduction in cells [338].

Microtubules, another anchoring point for the LINC complex, seem to have increased abnormal organization and catastrophe rate with advanced age [220, 221]. Similarly, in vitro studies conducted under both real and simulated microgravity conditions seem to disrupt the organization of microtubules and microtubule‐associated proteins [69, 180, 223, 224, 225, 226, 227, 228]. Papaseit et al. suggest that microtubules require gravity to self‐organise [223]. Moreover, Schatten et al. observed that microtubule disruption during clinorotation leads to mitochondrial clustering [224]. This clustering can increase the production of ROS [339], which has also been reported in multiple studies on simulated microgravity [47, 84, 85, 87, 88]. Elevated ROS further destabilizes microtubules [340], reinforcing a cycle of oxidative damage and cytoskeletal disorganization.

In addition to the disorganization of microtubules, actin filaments, which form another crucial part of the cytoskeleton, also exhibit changes in response to altered gravity and aging. Actin filaments tend to decrease in number and in mRNA levels under real and simulated microgravity [65, 196, 197, 198], whereas their numbers increase in various tissues during ageing [199, 200, 202]. Disruption of the cytoskeleton seems to alter nuclear shape and lamin organization, leading to changes in chromatin organization [341]. The chromatin landscape appears to play a role in the choice of DNA repair pathways [342]. Errors in this selection process may contribute to deficiencies in DNA repair mechanisms observed in both accelerated aging and under real and simulated microgravity [47, 48, 55, 56, 57]. These deficiencies may in turn result in the accumulation of DNA damage in both scenarios [42, 44, 45, 46, 47, 48, 49, 50, 51]. Furthermore, abnormal nuclear morphology, which is observed in aging [67, 68] as well as in real [69, 71] and simulated microgravity [51, 70, 72], can lead to clustering of nuclear pore complexes [343]. Such clustering may influence the kinetics of molecular transport across the nuclear envelope, including the import of DNA repair enzymes. In addition to obstructing physical access, the spatial arrangement of nuclear pore complexes has also been implicated in influencing DNA repair outcomes [344].

Mutations in the LMNA gene or the nuclear envelope‐associated Nesp1 protein alter the behavior of the mechanosensitive transcription factors YAP/TAZ [345]. Upon mechanical activation, YAP/TAZ translocate from the cytoplasm to the cell nucleus and change gene expression; depending on the cell type, this initiates different cell behavior [346]. YAP1 mRNA and protein levels seem to decrease with advanced age [282]. Under hypergravity conditions, YAP1 protein expression increases in hMEC‐1 cells [207]. In contrast, both real and simulated microgravity result in decreased YAP1 mRNA or protein levels in neonatal human cardiovascular progenitor cells and in the soleus and EDL muscles of rodents [57, 347]. Additionally, YAP1 expression in adult human cardiovascular progenitor cells appears time‐dependent, with levels initially rising before subsequently decreasing [26, 57]. During aging, YAP/TAZ translocation is cell‐specific. For fibroblasts and endothelial cells, YAP in the nucleus decreases, while for skeletal muscle cells, it accumulates [253, 268, 282]. Only a single article was found for altered gravity which reported increased YAP in the nucleus for a colorectal cancer cell line under simulated microgravity conditions [348]. Dupont et al. observed that YAP/TAZ translocation seems to respond to cytoskeletal tension generated by ECM stiffness and cell spreading [349]. The responsiveness to changes in the stiffness and adhesive area seems to alter with age, as it appears a more rigid ECM is required for the same activation [282]. Unfortunately, the responsiveness of YAP/TAZ is not investigated in altered gravity.

Responsiveness is a reoccurring topic in aging, which is ill‐investigated in altered gravity. With age, stimulus‐induced actin polymerization is reduced and the remodeling rate is decreased in mesenchymal stem cells and leukocytes [110, 350]. Although actin has been relatively well investigated in altered gravity compared to other components of cells, no studies were found that specifically focus on the response of the actin cytoskeleton to a biochemical stimulus under altered gravity conditions. Compromised actin remodeling is considered to also contribute to a weakened immune system [351]. Furthermore, aging appears to decrease the responsiveness of osteocytes to IGF‐1 [352]. With advanced age, mechanosensitivity appears to decline as fewer osteocytes respond to stimuli, their responses are delayed, and their Ca2+ signaling is diminished [353]. Similar observations were reported for cultured osteocytes in simulated microgravity [354], along with reduced responsiveness of osteoblasts to IGF‐1 in simulated microgravity and increased responsiveness in hypergravity [166].

Given that Piezo1 is a mechanosensitive ion channel involved in transducing mechanical forces into Ca^2+^ signals, it is a plausible contributor to the decline in calcium signaling and responsiveness. Its activity is modulated by the cytoskeleton, which normally buffers mechanical inputs. When this buffering is disrupted, such as by the reduction of actin filaments in real and simulated microgravity, Piezo1 becomes more sensitive, leading to increased calcium influx and promoting cytoskeletal remodeling. Paradoxically, excessive Piezo1 activation can destabilize the cytoskeleton, while its downregulation appears to stabilize actin filaments and reduce mechanical responsiveness [355]. This complex, bidirectional relationship may underlie conflicting findings of Piezo1 expression in altered gravity. It is upregulated in HUVECs under clinorotation [356] but downregulated in bone marrow‐derived mesenchymal stem cells during hindlimb unloading [357].

The ECM, another important element in mechanotransduction, is a support network consisting of, e.g., collagens, elastin, lamins, fibronectin, tenascins, growth factors and MMPs [246]. Collagen fibrils appear to have increased fragmentation with advanced age [235, 240, 241]. Articles seem to be inconclusive on the effect of gravity on collagen fibrils. Some state no change, while others observe a decrease in fibril size or fragmentation [32, 242, 243, 244, 245]. We expect that the duration of the experiments plays a critical role in these observations. No differences were observed for short‐duration rodent experiments in real microgravity (7 or 14 days) [32, 242], whereas longer experiments in both humans and rodents (30 days or 6 months) [243, 245] reported collagen fiber fragmentation and decreased fiber size. Bed rest simulated microgravity appears to have no effect on the collagen content even after 90 days of immobilization [358], while exposure to hypergravity at 3 g for 16 h disrupted collagen fibrils in mouse epiphyseal chondrocytes [244].

The expression of the precursor of collagen, procollagen, decreases with aging in the skin, while in real microgravity it increases [280, 359]. Neutelings et al. speculate that procollagen production is increased in mice on board the ISS due to an excessive early degradation of newly formed procollagen molecules [359]. The same behavior is observed for the precursor of elastin, tropoelastin, which also seems to decrease with age in the human aorta but increases in adipose‐derived stem cells under simulated microgravity conditions [360, 361].

Decreased collagen fiber size in murine tendon and bone could be caused by an increase in MMP expression [243]. MMPs break down the ECM, which is required for remodeling [246]. With age, there is an increase in expression for multiple MMPs [235, 241, 247]. We also see an increase in MMPs for real and simulated microgravity and hypergravity [207, 238, 248, 249, 362]. Some papers hypothesize that with advanced age, the change in MMP production causes collagen fibril fragmentation, which results in compromised adhesion of the cells to the ECM, and therefore the cells appear to spread less [240, 280]. The phenotype of less cell spreading is also observed in (simulated) microgravity [197, 230, 284]. In addition, as mentioned earlier, YAP/TAZ translocation appears to be affected by cell spreading [349].

In the current study, we limited our review to the 165 items related to biological aging at the level of the cell. Most aging‐related changes are reported from real or simulated microgravity studies using humans or translational animal models. These studies often include environmental issues like confinement, altered circadian rhythms, or, especially for orbital flights, increased exposure to ionizing radiation [8, 13, 363]. In the current study, we have tried to eliminate these confounding factors as much as possible in order to isolate only the effect of gravity. This broad scoping review provided a high‐level overview of the 165 aging‐related items related to altered gravity. Comparisons are made between different animal models or between in vivo and in vitro models. The work identifies some themes and items that should be researched in more detail in altered gravity conditions in order to better understand the aging‐related phenotypes in astronauts. Also, biological aging‐related effects seen at the level of the whole organism, organ, or tissue, such as changes in the microbiome, endocrinological changes, and reduced tissue characteristics like osteoporosis or sarcopenia, are not included in our review. Hence, the effects as reported in the literature on aging and spaceflight/microgravity, other than those that have been identified in this review, might have emerged from different environmental characteristics besides gravity.

To better understand the relationship between aging and gravity, besides more gravity research as well as having access to dedicated in‐flight instruments common in biophysical research [364], a better standardization of experiments would be beneficial. Simulated and real microgravity are often used interchangeably, while they do not by definition lead to the same results, as they do not provide the same conditions. Additionally, hypergravity is rarely used as a method to explore the effect of altered gravity, although it offers good and cost‐effective research opportunities based in the gravity continuum or the Reduced Gravity Paradigm [20, 21]. Furthermore, there is also a significant difference in the behavior of cell lines and cancer cells compared to primary cells. Given that cancer cells and other cell lines go through a process of immortalization, we suspect that they are not the best models for biological aging and gravity‐related research.

## 5 Conclusions

The body is a complex and well‐balanced system. It is resilient and can counteract many and diverse malfunctions. However, both biological aging and microgravity seem to induce an imbalance, resulting in many phenotypes seen not only for higher‐level systems but also, to some extent, from a cellular point of view. Based on the 11 themes and 165 identified items related to aging used as the baseline for this comprehensive study, approximately 15% of the items are not in agreement between biological aging and microgravity, and another 17% are ambiguous when evaluated from a cellular perspective. We speculate that mechanotransduction is key in the resemblance of phenotypes seen in biological aging and microgravity. Gravity research is lacking studies directed toward the many components making up the mechanical link that facilitates cellular mechanoperception and mechanotransduction. More research on this topic would not only enhance our understanding of basic cellular processes but also help to support future astronauts and shed light on the fundamentals of biological aging.

## Author Contributions

**Sharon van Rijthoven:** conceptualization, acquisition of data, and writing (manuscript, review and editing). **Jack J. W. A. van Loon:** conceptualization, acquisition of funding, supervision, and writing (review and editing).

## Conflicts of Interest

The authors declare no conflicts of interest.

## Supporting information


Data S1.

## Data Availability

The data that support the findings of this study are available in the Supporting Information of this article.
