# Supplementary material for: Aging and Altered Gravity: A Cellular Perspective
Source: FASEB J. 2025 Jun 27;39(13):e70777. doi: 10.1096/fj.202402989R (PMC12204310; doi:10.1096/fj.202402989R)
Supplement: Supplementary file 1 — Data S1. [file FSB2-39-e70777-s001.pdf]

Supplemental Material

Table of Contents

Figure S1: Decision tree for selecting altered gravity-related articles 2
Table S1 Description 3
Table S1 4

Figure S1: Decision tree for selecting altered gravity-related articles

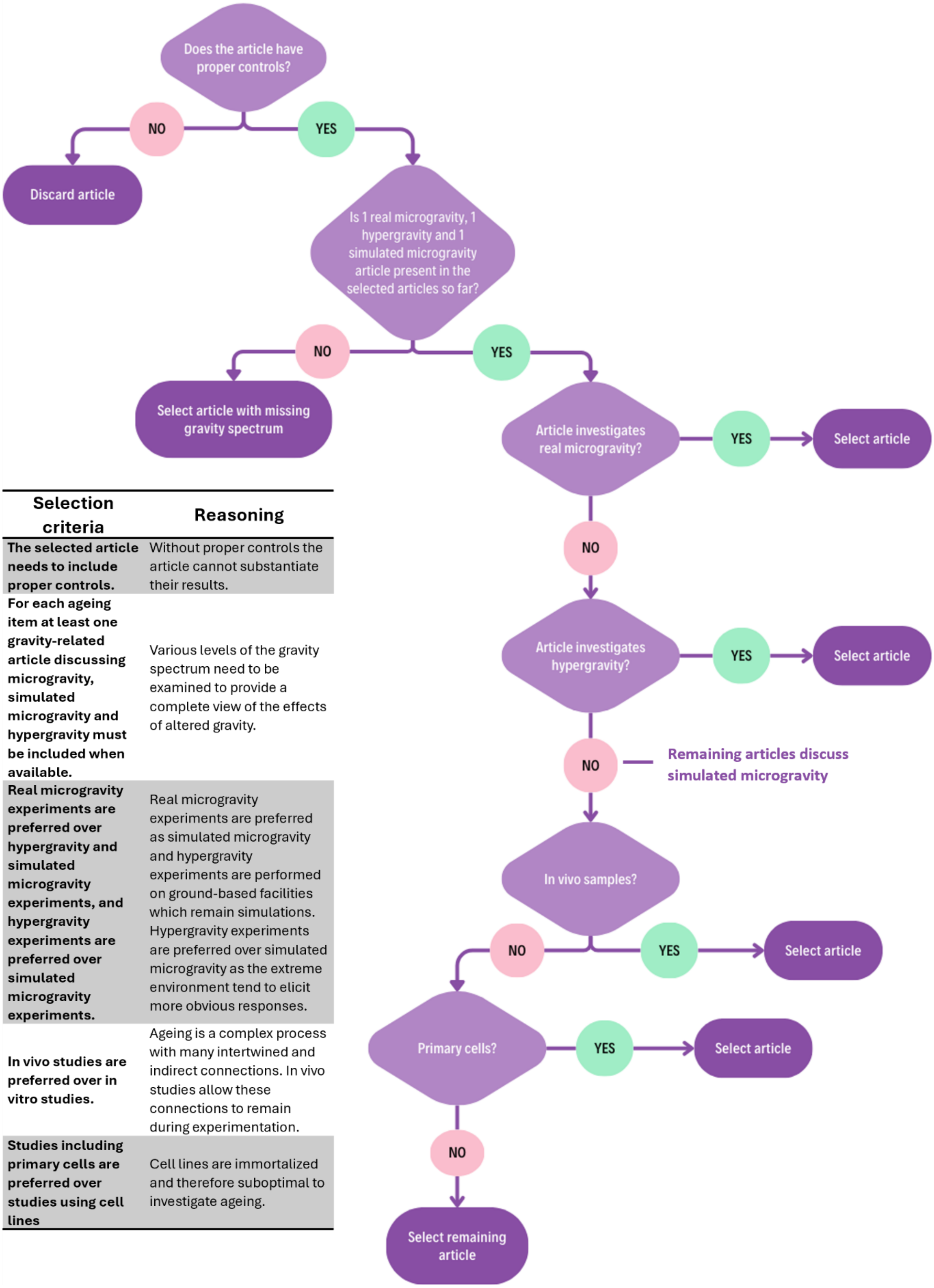

Selection criteria	Reasoning
**The selected article needs to include proper controls.**	Without proper controls the article cannot substantiate their results.
**For each ageing item at least one gravity-related article discussing microgravity, simulated microgravity and hypergravity must be included when available.**	Various levels of the gravity spectrum need to be examined to provide a complete view of the effects of altered gravity.
**Real microgravity experiments are preferred over hypergravity and simulated microgravity experiments, and hypergravity experiments are preferred over simulated microgravity experiments.**	Real microgravity experiments are preferred as simulated microgravity and hypergravity experiments are performed on ground-based facilities which remain simulations. Hypergravity experiments are preferred over simulated microgravity as the extreme environment tend to elicit more obvious responses.
**In vivo studies are preferred over in vitro studies.**	Ageing is a complex process with many intertwined and indirect connections. In vivo studies allow these connections to remain during experimentation.
**Studies including primary cells are preferred over studies using cell lines**	Cell lines are immortalized and therefore suboptimal to investigate ageing.

Figure S1. *The decision tree for selecting altered gravity-related articles when more than five relevant articles were found for a specific biological ageing sign. The table provides reasoning for the selection criteria in the decision tree.*

Table S1 Description

Sheets

Each sheet of excel document represents one of the eleven arbitrary categorised themes: DNA & epigenetics, Mitochondria, Nucleus, Immune system, Protein & metabolism, Lysosome & degradation, Cell cycle, Cytoskeleton, Extracellular Matrix (ECM), Cell mechanics, Cell signalling

Columns

On the first row a description for each column can be found which is the same for each sheet.

- Column A: Describes the investigated sign of ageing which is obtained from the works on biological ageing from López-Otín C. et al. (2013), Schmauck-Medina T. et al. (2022), Bajpai A. et al. (2021), Phillip J. M. et al. (2015), and Starodubtseva M. (2011)
- Column B: Describes the effect of ageing on the sign which is obtained from the works or the referred material from López-Otín C. et al. (2013), Schmauck-Medina T. et al. (2022), Bajpai A. et al. (2021), Phillip J. M. et al. (2015), and Starodubtseva M. (2011).
- Column C: Describes the effect of altered gravity on the ageing sign. Numbers behind the observations between parenthesis refer to the references given in Column D. Observations are categorised per type of altered gravity:
  MG = real microgravity; orbital spaceflight, sounding rockets, and parabolic flight
  SMG = simulated microgravity; clinorotation, random positioning machine, (head down tilt) bed rest, dry immersion, and hindlimb unloading
  HG = hypergravity
  A maximum of five articles is selected from literature. The selection procedure is described in Figure 2 of the article. Anything less than five articles indicates that only the given references were found with the described search term, data bases and in the assigned search period.
- Column D: References of the articles investigating the ageing sign in altered gravity.

Colour coding

The items in Column A are colour coded in the same colours as shown in Figure 3. The colour depends on the majority of the outcome for the found results and the shade indicates the number of articles. An overview of the colours and shades is given below:

colour

The item acts similar between ageing and (simulated) microgravity and opposite between ageing and hypergravity.

The item acts opposite between ageing and (simulated) microgravity and similar between ageing and hypergravity.

For this item there was no clear majority for similar, opposite or no change in behaviour.

The item changes during ageing but seems not affected by altered gravity.

The item has not been covered in altered gravity research, but which is a characteristic in ageing.

shade

Five or more articles researched this topic and support this relationship

Less than five articles researched this topic and support this relationship

Table S1

DNA & EPIGENETICS			
**Item**	**Effect of ageing**	**Effect of altered gravity** (**MG** = real microgravity, **HG** = hypergravity, **SMG** = simulated microgravity)	**References**
DNA damage	DNA damage accumulation increases	**SMG**: increases(1,2), increases but only when DNA repair proteins are knocked out (3), increases for single strand break (4), increases but after 32h back to control values (5).	1. Singh R, Rajput M, Singh RP. Simulated microgravity triggers DNA damage and mitochondria-mediated apoptosis through ROS generation in human promyelocytic leukemic cells. Mitochondrion. 2021;61:114-124. doi:10.1016/j.mito.2021.09.006<br>2. Kumari R, Singh KP, DuMond JW. Simulated microgravity decreases DNA repair capacity and induces DNA damage in human lymphocytes. Journal of Cellular Biochemistry. 2009;107(4):723-731.<br>3. Li N, An L, Hang H. Increased sensitivity of DNA damage response-deficient cells to stimulated microgravity-induced DNA lesions. PLOS ONE. 2015;10(4). doi:10.1371/journal.pone.0125236<br>4. Roberts JE, Kukielczak BM, Chignell CF, Sik BH, Hu DN, Principato MA. Simulated microgravity induced damage in human retinal pigment epithelial cells. Mol Vis. 2006;12:633-638.<br>5. Uva BM, Masini MA, Sturla M, et al. Clinorotation-induced weightlessness influences the cytoskeleton of glial cells in culture. Brain Res. 2002;934(2):132-139.
Laminopathies	premature ageing	/	
Somatic mutations	accumulate with age	**MG**: increased for tp53 gene in astronauts compared to civilians(1)	1. Brojakowska A, Kour A, Thel MC, et al. Retrospective analysis of somatic mutations and clonal hematopoiesis in astronauts. Communications Biology. 2022;5(1). doi:10.1038/s42003-022-03777-z
Chromosomal aneuploidies	accumulates in a chromosome-specific manner	/	
Chromosomal copy number variations	accumulates in a chromosome-specific manner	/	
Chromosomal anomalies	Clonal mosaicism for large chromosomal anomalies increases	**MG**: Clonal hematopoiesis detected in 2 astronauts at a 20 year younger age compared to mean age it occurs(1)<br>**SMG**: no effect(2)	1. Mencia-Trinchant N, MacKay MJ, Chin C, et al. Clonal hematopoiesis before, during, and after human spaceflight. Cell Reports. 2020;33(10):108458. doi:10.1016/j.celrep.2020.108458<br>2. Wei L, Liu C, Kang L, et al. Experimental study on effect of simulated microgravity on structural chromosome instability of human peripheral blood lymphocytes. PLoS ONE. 2014;9(6). doi:10.1371/journal.pone.0100595
DNA repair	Deficiencies in DNA repair mechanisms cause accelerated ageing	**MG:**increased for HR, NER, DSB(1)<br>**SMG:**decreased expression of DNA repair gene BER, NER, MMR(2), decreased mRNA expression of BER, NER, MMR, but increased for NHEJ and HR(3), decreased DNA repair(4), decreased expression in genes for BER,NER, MMR,DSB(5).	1. Baio J, Martinez AF, Silva I, et al. Cardiovascular progenitor cells cultured aboard the International Space Station exhibit altered developmental and functional properties. npj Microgravity. 2018;4(1):13.<br>2. Kumari R, Singh KP, Dumond JW, Jr. Simulated microgravity decreases DNA repair capacity and induces DNA damage in human lymphocytes. J Cell Biochem. 2009;107(4):723-731.<br>3. Singh R, Rajput M, Singh RP. Simulated microgravity triggers DNA damage and mitochondria-mediated apoptosis through ROS generation in human promyelocytic leukemic cells. Mitochondrion. 2021;61:114-124. doi:10.1016/j.mito.2021.09.006<br>4. Degan P, Sancandi M, Zunino A, et al. Exposure of human lymphocytes and lymphoblastoid cells to simulated microgravity strongly affects energy metabolism and DNA repair. J Cell Biochem. 2005;94(3):460-469.<br>5. Zhao T, Tang X, Umeshappa CS, et al. Simulated Microgravity Promotes Cell Apoptosis Through Suppressing Uev1A/TICAM/TRAF/NF-κB-Regulated Anti-Apoptosis and p53/PCNA- and ATM/ATR-Chk1/2-Controlled DNA-Damage Response Pathways. J Cell Biochem. 2016;117(9):2138-2148.

Telomere length	shorten with age	\	
Telomerase	Adding or reactivating telomerase causes delayed ageing	**SMG:** telomerase activity decreased(1), telomerase activity increased(2).	1. Sun L, Gan B, Fan Y, Xie T, Hu Q, Zhuang F. Simulated microgravity alters multipotential differentiation of rat mesenchymal stem cells in association with reduced telomerase activity. Acta Astronautica. 2008;63(7-10):968-973. doi:10.1016/j.actaastro.2007.12.008<br>2. Fuentes TI, Appleby N, Raya M, et al. Simulated microgravity exerts an age-dependent effect on the differentiation of cardiovascular progenitors isolated from the human heart. PLOS ONE. 2015;10(7). doi:10.1371/journal.pone.0132378
H4K20 trimethylation	increases	\	
H3K4 trimethylation	change in landscape (in the brain)	**MG:** downregulated(1)<br>**SMG:** increased for IIx and IIb muscle fiber and no change for I and IIa myosin heavy chain(2)	1. Vahlensieck C, Thiel CS, Christoffel S, et al. Rapid downregulation of H3K4me3 binding to immunoregulatory genes in altered gravity in primary human M1 macrophages. International Journal of Molecular Sciences. 2022;24(1):603. doi:10.3390/ijms24010603<br>2. Pandorf CE, Haddad F, Wright C, Bodell PW, Baldwin KM. Differential epigenetic modifications of histones at the myosin heavy chain genes in fast and slow skeletal muscle fibers and in response to muscle unloading. American Journal of Physiology-Cell Physiology. 2009;297(1). doi:10.1152/ajpcell.00075.2009
H3K27 trimethylation	decreases	**MG:** increased(1)<br>**HG:** reduced(2)	1. Higashitani A, Hashizume T, Takiura M, et al. Histone deacetylase HDA-4-mediated epigenetic regulation in space-flown C. elegans. npj Microgravity. 2021;7(1). doi:10.1038/s41526-021-00163-7<br>2. Calcagno G, Ouzren N, Kaminski S, Ghislin S, Frippiat J-P. Chronic hypergravity induces a modification of histone H3 lysine 27 trimethylation at tcrβ locus in murine thymocytes. International Journal of Molecular Sciences. 2022;23(13):7133. doi:10.3390/ijms23137133
NuRD complex	diminished	\	
Heterochromatin	loss and redistribution of global and peripheral heterochromatin	\	
Heterochromatin Protein 1	decrease and overexpression extends longevity	\	

Transcriptional noise	increases	\	
H3K9me3	decreases	**SMG**: increases(1)	1. Li B, Zhao J, Ma J, et al. Cross-talk Between Histone and DNA Methylation Mediates Bone Loss in Hind Limb Unloading. J Bone Miner Res. 2021;36(5):956-967. doi:10.1002/jbmr.4253
Activator Protein 1	elavated	**SMG**: transactivation reduces(1,2), activation is blocked(3), expression is increased(4)	1. Ontiveros C, McCabe LR. Simulated microgravity suppresses osteoblast phenotype, Runx2 levels and AP-1 transactivation. J Cell Biochem. 2003;88(3):427-437. doi:10.1002/jcb.10410<br>2. Wang YC, Zhang S, Du TY, Wang B, Sun XQ. Clinorotation upregulates inducible nitric oxide synthase by inhibiting AP-1 activation in human umbilical vein endothelial cells. J Cell Biochem. 2009;107(2):357-363. doi:10.1002/jcb.22134<br>3. Morrow MA. Clinorotation differentially inhibits T-lymphocyte transcription factor activation. In Vitro Cell Dev Biol Anim. 2006;42(5-6):153-158. doi:10.1290/0601011.1<br>4. Granet C, Vico AG, Alexandre C, Lafage-Proust MH. MAP and src kinases control the induction of AP-1 members in response to changes in mechanical environment in osteoblastic cells. Cell Signal. 2002;14(8):679-688. doi:10.1016/s0898-6568(02)00008-6
DNA methylation	total genomic DNA methylation decreases	**MG:** increases(1), no change(2)<br>**SMG:** decreases(3), no change(4), altered(5)	1. Ogneva IV, Loktev SS, Sychev VN. Cytoskeleton structure and total methylation of mouse cardiac and lung tissue during space flight. PLoS One. 2018;13(5):e0192643. Published 2018 May 16. doi:10.1371/journal.pone.0192643<br>2. Garrett-Bakelman FE, Darshi M, Green SJ, et al. The NASA Twins Study: A multidimensional analysis of a year-long human spaceflight. Science. 2019;364(6436):eaau8650. doi:10.1126/science.aau8650<br>3. Singh KP, Kumari R, Dumond JW. Simulated microgravity-induced epigenetic changes in human lymphocytes. J Cell Biochem. 2010;111(1):123-129. doi:10.1002/jcb.22674<br>4. Usik MA, Ogneva IV. DNA methylation in mouse spermatozoa under long-term modeling the effects of microgravity. Russ J Dev Biol. 2019;50(4):216-224. doi:10.1134/S1062360419040076<br>5. Chowdhury B, Seetharam A, Wang Z, et al. A Study of Alterations in DNA Epigenetic Modifications (5mC and 5hmC) and Gene Expression Influenced by Simulated Microgravity in Human Lymphoblastoid Cells. PLoS One. 2016;11(1):e0147514. Published 2016 Jan 28. doi:10.1371/journal.pone.0147514
Hyperploid cell	accumulate in tissue	\	
DNA damage at telomere	more damage induces senescence and/or apoptosis	\	

MITOCHONDRIA			
**Item**	**Effect of ageing**	**Effect of altered gravity** (**MG** = real microgravity, **HG** = hypergravity, **SMG** = simulated microgravity)	**References**
Mutations and deletions in mtDNA	accumulate	**SMG:** no deletions detected between before and after treatment(1)	1. Pesce V, Cormio A, Fracasso F, Lezza AM, Cantatore P, Gadaleta MN. Rat hindlimb unloading: Soleus and Extensor Digitorum Longus histochemistry, mitochondrial DNA content and mitochondrial DNA deletions. Biosci Rep. 2002;22(1):115-125. doi:10.1023/a:1016069208073
Reactive Oxygen Species	increases	**SMG**: increased after ~9 hours(1), ~24 hours(2), ~48 hours(3), ~72 hours(4), ~24-96 hours(5)	1. Manis C, Manca A, Murgia A, et al. Understanding the Behaviour of Human Cell Types under Simulated Microgravity Conditions: The Case of Erythrocytes. Int J Mol Sci. 2022;23(12):6876. Published 2022 Jun 20. doi:10.3390/ijms23126876<br>2. Zhang Y, Wang H, Lai C, Wang L, Deng Y. Comparative proteomic analysis of human SH-SY5Y neuroblastoma cells under simulated microgravity. Astrobiology. 2013;13(2):143-150. doi:10.1089/ast.2012.0822<br>3. Acharya A, Nemade H, Papadopoulos S, et al. Microgravity-induced stress mechanisms in human stem cell-derived cardiomyocytes. iScience. 2022;25(7):104577. Published 2022 Jun 11. doi:10.1016/j.isci.2022.104577<br>4. Singh R, Rajput M, Singh RP. Simulated microgravity triggers DNA damage and mitochondria-mediated apoptosis through ROS generation in human promyelocytic leukemic cells. Mitochondrion. 2021;61:114-124. doi:10.1016/j.mito.2021.09.006<br>5. Morabito C, Guarnieri S, Cucina A, Bizzarri M, Mariggiò MA. Antioxidant Strategy to Prevent Simulated Microgravity-Induced Effects on Bone Osteoblasts. Int J Mol Sci. 2020;21(10):3638. Published 2020 May 21. doi:10.3390/ijms21103638
DNA Polymerase γ	deficient DNA Polymerase γ causes accelerated ageing	/	
Mitochondrial bioenergetics	decreased efficiency	**SMG**: increased mitochondrial bioenergetics(1), impaired mitochondrial energy potential(2)	1. Espinosa-Jeffrey A, Nguyen K, Kumar S, et al. Simulated microgravity enhances oligodendrocyte mitochondrial function and lipid metabolism. J Neurosci Res. 2016;94(12):1434-1450. doi:10.1002/jnr.23958<br>2. Michaletti A, Gioia M, Tarantino U, Zolla L. Effects of microgravity on osteoblast mitochondria: a proteomic and metabolomics profile. Sci Rep. 2017;7(1):15376. Published 2017 Nov 13. doi:10.1038/s41598-017-15612-1
Mitochondrial uncoupling	increase uncoupling extends lifespan	**SMG**: increased(1)	1. Maccarrone M, Battista N, Meloni M, et al. Creating conditions similar to those that occur during exposure of cells to microgravity induces apoptosis in human lymphocytes by 5-lipoxygenase-mediated mitochondrial uncoupling and cytochrome c release. J Leukoc Biol. 2003;73(4):472-481. doi:10.1189/jlb.0602295

Steroidogenic acute regulatory protein	decreases	/	
Mitochondrial biogenesis	decreases	**SMG**: decreased(1,2,3,4), increased(5)	1. Lvova ID, Sharlo KA, Rozhkov SV, et al. The role of glycogen synthase kinase 3 activity in the regulation of mitochondrial biogenesis in rat postural muscle under hindlimb unloading. Biochem Moscow Suppl Ser A. 2021;15(6):372-377. doi:10.1134/S1990747821060076.<br>2. Liu J, Peng Y, Cui Z, et al. Depressed mitochondrial biogenesis and dynamic remodeling in mouse tibialis anterior and gastrocnemius induced by 4-week hindlimb unloading. IUBMB Life. 2012;64(11):901-910. doi:10.1002/iub.1087<br>3. Rosa-Caldwell ME, Brown JL, Perry RA Jr, et al. Regulation of mitochondrial quality following repeated bouts of hindlimb unloading. Appl Physiol Nutr Metab. 2020;45(3):264-274. doi:10.1139/apnm-2019-0218<br>4. Liu J, Peng Y, Feng Z, et al. Reloading functionally ameliorates disuse-induced muscle atrophy by reversing mitochondrial dysfunction, and similar benefits are gained by administering a combination of mitochondrial nutrients. Free Radic Biol Med. 2014;69:116-128. doi:10.1016/j.freeradbiomed.2014.01.003<br>5. Tan X, Xu A, Zhao T, et al. Simulated microgravity inhibits cell focal adhesions leading to reduced melanoma cell proliferation and metastasis via FAK/RhoA-regulated mTORC1 and AMPK pathways. Sci Rep. 2018;8:3769. doi:10.1038/s41598-018-20459-1.
PGC-1α	overexpression extends lifespan	**MG:** decreased(1)<br>**SMG:** decreased(1,2,3,4,5)	1. Camerino GM, Pierno S, Liantonio A, et al. Effects of pleiotrophin overexpression on mouse skeletal muscles in normal loading and in actual and simulated microgravity. PLoS One. 2013;8(8):e72028. Published 2013 Aug 28. doi:10.1371/journal.pone.0072028<br>2. Liu J, Peng Y, Cui Z, et al. Depressed mitochondrial biogenesis and dynamic remodeling in mouse tibialis anterior and gastrocnemius induced by 4-week hindlimb unloading. IUBMB Life. 2012;64(11):901-910. doi:10.1002/iub.1087<br>3. Rosa-Caldwell ME, Brown JL, Perry RA Jr, et al. Regulation of mitochondrial quality following repeated bouts of hindlimb unloading. Appl Physiol Nutr Metab. 2020;45(3):264-274. doi:10.1139/apnm-2019-0218<br>4. Irimia JM, Guerrero M, Rodriguez-Miguelez P, et al. Metabolic adaptations in skeletal muscle after 84 days of bed rest with and without concurrent flywheel resistance exercise. J Appl Physiol (1985). 2017;122(1):96-103. doi:10.1152/japplphysiol.00521.2016<br>5. Cannavino J, Brocca L, Sandri M, Bottinelli R, Pellegrino MA. PGC1-α over-expression prevents metabolic alterations and soleus muscle atrophy in hindlimb unloaded mice. J Physiol. 2014;592(20):4575-4589. doi:10.1113/jphysiol.2014.275545

CELL CYCLE			
**Item**	**Effect of ageing**	**Effect of altered gravity** (**MG** = real microgravity, **HG** = hypergravity, **SMG** = simulated microgravity)	**References**
Nuclear lamina gene	mutations in gene cause accelerated ageing	/	
Prelamin A	abberent production cause accelerated ageing, progerin accumulates with age	/	
Lamin B1	decreases in senecent cells	**SMG**: decreased(1)	1. Touchstone H, Byrd R, Loisate S, et al. Recovery of stem cell proliferation by low intensity vibration under simulated microgravity requires LINC complex. npj Microgravity. 2019;5:11. doi:10.1038/s41526-019-0072-5.
Maturation mRNA	genes involved are upregulated	/	
Alternative splicing	altered isoform ratios and splicing factor expression	**MG:** altered(1)<br>**SMG:** altered(2)	1. Henrich M, Ha P, Wang Y, et al. Alternative splicing diversifies the skeletal muscle transcriptome during prolonged spaceflight. Skelet Muscle. 2022;12(1):11. Published 2022 May 31. doi:10.1186/s13395-022-00294-9<br>2. Sun J, Yang H, Yang X, et al. Global alternative splicing landscape of skeletal muscle atrophy induced by hindlimb unloading. Ann Transl Med. 2021;9(8):643. doi:10.21037/atm-20-5388
Polyadenylation of mRNA	altered	/	
YAP/TAZ responsiveness	altered and higher tresshold before a response	/	

YAP location	less in the cell nucleus for fibroblasts, adipose and lung tissue, more in the cell nucleus voor skeletal muscle	**SMG**: more in the nucleus for colorectal cancer cell(1)	1. Arun RP, Sivanesan D, Patra B, Varadaraj S, Verma RS. Simulated microgravity increases polyploid giant cancer cells and nuclear localization of YAP. Sci Rep. 2019;9(1):10684. Published 2019 Jul 23. doi:10.1038/s41598-019-47116-5
YAP1 mrna, protein and phosphorylation	mrna and protein levels decreased, phosphorylation increased	**MG:** decreased mRNA in neonatal cells(1), initially increased mRNA in adult cells but decreases again(1,2)<br>**HG:** increased YAP1 protein(3)<br>**SMG:** initially increased mRNA in adult cells but decreases again(2), decreased protein and no change in phosphorylation (4)	1. Baio J, Martinez AF, Silva I, et al. Cardiovascular progenitor cells cultured aboard the International Space Station exhibit altered developmental and functional properties. npj Microgravity. 2018;4:13. doi:10.1038/s41526-018-0048-x.<br>2. Camberos V, Baio J, Bailey L, Hasaniya N, Lopez LV, Kearns-Jonker M. Effects of Spaceflight and Simulated Microgravity on YAP1 Expression in Cardiovascular Progenitors: Implications for Cell-Based Repair. Int J Mol Sci. 2019;20(11):2742. Published 2019 Jun 4. doi:10.3390/ijms20112742<br>3. De Cesari C, Barravecchia I, Pyankova OV, et al. Hypergravity Activates a Pro-Angiogenic Homeostatic Response by Human Capillary Endothelial Cells. Int J Mol Sci. 2020;21(7):2354. Published 2020 Mar 28. doi:10.3390/ijms21072354<br>4. Usuki F, Fujimura M, Nakamura A, Nakano J, Okita M, Higuchi I. Local Vibration Stimuli Induce Mechanical Stress-Induced Factors and Facilitate Recovery From Immobilization-Induced Oxidative Myofiber Atrophy in Rats. Front Physiol. 2019;10:759. Published 2019 Jun 20. doi:10.3389/fphys.2019.00759
SRF protein	decreases	**MG:** gene is downregulated(1)<br>**SMG:** gene is downregulated(2), reduced mRNA and protein(3), no change in protein (4), gene is upregulated(5)	1. Chang TT, Walther I, Li CF, et al. The Rel/NF-κB pathway and transcription of immediate early genes in T cell activation are inhibited by microgravity. J Leukoc Biol. 2012;92(6):1133-1145. doi:10.1189/jlb.0312157<br>2. Xu D, Guo YB, Zhang M, Sun YQ. The subsequent biological effects of simulated microgravity on endothelial cell growth in HUVECs. Chin J Traumatol. 2018;21(4):229-237. doi:10.1016/j.cjtee.2018.04.004<br>3. Giger JM, Bodell PW, Zeng M, Baldwin KM, Haddad F. Rapid muscle atrophy response to unloading: pretranslational processes involving MHC and actin. J Appl Physiol (1985). 2009;107(4):1204-1212. doi:10.1152/japplphysiol.00344.2009<br>4. Sakuma K, Watanabe K, Hotta N, et al. The adaptive responses in several mediators linked with hypertrophy and atrophy of skeletal muscle after lower limb unloading in humans. Acta Physiol (Oxf). 2009;197(2):151-159. doi:10.1111/j.1748-1716.2009.01995.x<br>5. Rojo-García AV, Vanmunster M, Pacolet A, Suhr F. Physical inactivity by tail suspension alters markers of metabolism, structure, and autophagy of the mouse heart. Physiol Rep. 2023;11(2):e15574. doi:10.14814/phy2.15574
LAP2	reduces	**/**	
Nucleoplasmic Lamin A/C	decreases	/	
Lamin A/C location	for young throughout the nucleoplasm, for old at the nuclear rim	/	

Nuclei shape	more abnormal shape (folds, stretching, lobs, fragmentation)	**MG:** more abnormal shape (1), more oblong shape (2)<br>**SMG:** more abnormal shape(3),decreased nucleir height (4),less round shape (5)	1. Nabavi N, Khandani A, Camirand A, Harrison RE. Effects of microgravity on osteoclast bone resorption and osteoblast cytoskeletal organization and adhesion. Bone. 2011;49(5):965-974. doi:10.1016/j.bone.2011.07.036<br>2. Hughes-Fulford M, Tjandrawinata R, Fitzgerald J, Gasuad K, Gilbertson V. Effects of microgravity on osteoblast growth. Gravit Space Biol Bull. 1998;11(2):51-60.<br>3. Uva BM, Masini MA, Sturla M, et al. Clinorotation-induced weightlessness influences the cytoskeleton of glial cells in culture. Brain Res. 2002;934(2):132-139. doi:10.1016/s0006-8993(02)02415-0<br>4. Neelam S, Richardson B, Barker R, et al. Changes in Nuclear Shape and Gene Expression in Response to Simulated Microgravity Are LINC Complex-Dependent. Int J Mol Sci. 2020;21(18):6762. Published 2020 Sep 15. doi:10.3390/ijms21186762<br>5. Dai TX, Son HN, Chi HNQ, et al. Simulated Microgravity Induces the Proliferative Inhibition and Morphological Changes in Porcine Granulosa Cells. Curr Issues Mol Biol. 2021;43(3):2210-2219. Published 2021 Dec 10. doi:10.3390/cimb43030155
Nuclear pores	clustering	/	
Nuclear lamina shape	more abnormal shape	/	

IMMUNE SYSTEM			
**Item**	**Effect of ageing**	**Effect of altered gravity**<br>(**MG** = real microgravity, **HG** = hypergravity, **SMG** = simulated microgravity)	**References**
Inflammation genes	overexpression increases with age	**MG:** immune & inflammation related gene expression were grossly downregulated(1), no change(2)<br>**HG:** some inflammation related genes downregulated(3)<br>**SMG:** inflammation related genes upregulated(2,4,5)	1. Holley JM, Stanbouly S, Pecaut MJ, Willey JS, Delp M, Mao XW. Characterization of gene expression profiles in the mouse brain after 35 days of spaceflight mission. NPJ Microgravity. 2022;8(1):35. Published 2022 Aug 10. doi:10.1038/s41526-022-00217-4<br>2. Han Y, Zeger L, Tripathi R, et al. Molecular genetic analysis of neural stem cells after space flight and simulated microgravity on earth. Biotechnol Bioeng. 2021;118(10):3832-3846. doi:10.1002/bit.27858<br>3. Morbidelli L, Marziliano N, Basile V, et al. Effect of hypergravity on endothelial cell function and gene expression. Microgravity Sci Technol. 2009;21(1):135-140. doi:10.1007/s12217-008-9067-7.<br>4. Lu SY, Guo S, Chai SB, et al. Proteomic analysis of the effects of simulated microgravity in human gastric mucosal cells. Life Sci Space Res (Amst). 2022;32:26-37. doi:10.1016/j.lssr.2021.10.001<br>5. Chai S, Guo S, Yang J, et al. Inflammation is involved in response of gastric mucosal epithelial cells under simulated microgravity by integrated transcriptomic analysis. Am J Transl Res. 2021;13(8):9195-9207. Published 2021 Aug 15.
Inflammaging	increased	/	
NF-κB	activation increases, inhibition of NF-κB increases longevity	**MG:** up-regulation (NFKB1, NFKB3, NFKBIA and NFKBIB) and reduced protein content of NF-κB p65(1), NFκB signaling pathway genes downregulated and nfkb1 is upregulated(2), downregulated c-Rel, RELA,NF-κB1(3)<br>**HG:** upregulated NFKBIA(1)<br>**SMG:** no change(1), deNFκB signaling pathway genes downregulated and nfkb1 is upregulated(2), creased activation(4), reduced pNF-κB and translocation from nucleus to cytoplasm(5)	1. Nassef MZ, Kopp S, Melnik D, et al. Short-Term Microgravity Influences Cell Adhesion in Human Breast Cancer Cells. Int J Mol Sci. 2019;20(22):5730. Published 2019 Nov 15. doi:10.3390/ijms20225730<br>2. Shi L, Tian H, Wang P, et al. Spaceflight and simulated microgravity suppresses macrophage development via altered RAS/ERK/NFκB and metabolic pathways. Cell Mol Immunol. 2021;18(6):1489-1502. doi:10.1038/s41423-019-0346-6<br>3. Chang TT, Walther I, Li CF, et al. The Rel/NF-κB pathway and transcription of immediate early genes in T cell activation are inhibited by microgravity. J Leukoc Biol. 2012;92(6):1133-1145. doi:10.1189/jlb.0312157<br>4. Calcagno G, Jeandel J, Frippiat JP, Kaminski S. Simulated Microgravity Disrupts Nuclear Factor κB Signaling and Impairs Murine Dendritic Cell Phenotype and Function. Int J Mol Sci. 2023;24(2):1720. Published 2023 Jan 15. doi:10.3390/ijms24021720<br>5. Zhao T, Li R, Tan X, et al. Simulated Microgravity Reduces Focal Adhesions and Alters Cytoskeleton and Nuclear Positioning Leading to Enhanced Apoptosis via Suppressing FAK/RhoA-Mediated mTORC1/NF-κB and ERK1/2 Pathways. Int J Mol Sci. 2018;19(7):1994. Published 2018 Jul 8. doi:10.3390/ijms19071994
Inflammatory mediators	increased	**MG:** increased TNFα, IL-8, IL-1ra, no effect for IL-1α, IL-1β, IL-2, IFN-γ, IL-17, IL-4, IL-5, IL-10(1), increased IFN-γ, also a trend in other mediators being upregulated but not significant(2)<br>**SMG:** no change for many mediators, but reduced IL-6(3), little to no change, only IL-8 was increased and VEGF(4), decreased levels in response to harmfull agents for IL-2 and IFN-γ, decreased or no change for IL-10 and TNFα(5)	1. Crucian BE, Zwart SR, Mehta S, et al. Plasma cytokine concentrations indicate that in vivo hormonal regulation of immunity is altered during long-duration spaceflight. J Interferon Cytokine Res. 2014;34(10):778-786. doi:10.1089/jir.2013.0129<br>2. Chang TT, Spurlock SM, Candelario TL, Grenon SM, Hughes-Fulford M. Spaceflight impairs antigen-specific tolerance induction in vivo and increases inflammatory cytokines. FASEB J. 2015;29(10):4122-4132. doi:10.1096/fj.15-275073<br>3. Cotrupi S, Ranzani D, Maier JA. Impact of modeled microgravity on microvascular endothelial cells. Biochim Biophys Acta. 2005;1746(2):163-168. doi:10.1016/j.bbamcr.2005.10.002<br>4. Ratushnyy A, Yakubets D, Andreeva E, et al. Simulated microgravity modulates the mesenchymal stromal cell response to inflammatory stimulation. Sci Rep. 2019;9:9279. doi:10.1038/s41598-019-45741-8.<br>5. Van Walleghem M, Tabury K, Fernandez-Gonzalo R, et al. Gravity-Related Immunological Changes in Human Whole Blood Cultured Under Simulated Microgravity Using an In Vitro Cytokine Release Assay. J Interferon Cytokine Res. 2017;37(12):531-540. doi:10.1089/jir.2017.0065

M1 macrophages	increased	/	
M2 macrophages	decreased in cardiac tissue, increased in bone marrow	/	
CD14	decreased	**MG:** reduced(1,2), no change or reduced depending on the direction of the gravity vector for the 1g controls(3)<br>**SMG:** no change(4), slightly reduced(5)	1. Crucian BE, Cubbage ML, Sams CF. Altered cytokine production by specific human peripheral blood cell subsets immediately following space flight. J Interferon Cytokine Res. 2000;20(6):547-556. doi:10.1089/10799900050044741<br>2. Kaur I, Simons ER, Kapadia AS, Ott CM, Pierson DL. Effect of spaceflight on ability of monocytes to respond to endotoxins of gram-negative bacteria. Clin Vaccine Immunol. 2008;15(10):1523-1528. doi:10.1128/CVI.00065-08<br>3. Tauber S, Lauber BA, Paulsen K, et al. Cytoskeletal stability and metabolic alterations in primary human macrophages in long-term microgravity. PLoS One. 2017;12(4):e0175599. Published 2017 Apr 18. doi:10.1371/journal.pone.0175599<br>4. Pao SI, Chien KH, Lin HT, Tai MC, Chen JT, Liang CM. Effect of microgravity on the mesenchymal stem cell characteristics of limbal fibroblasts. J Chin Med Assoc. 2017;80(9):595-607. doi:10.1016/j.jcma.2017.01.008<br>5. Tang C, Zheng Y, Wang T, et al. Effects of weightlessness on expression of TLR4/CD14 and chemotactic factors in gingival tissue of rhesus macaques. Int J Clin Exp Pathol. 2016;9(8):8162-8171.
Bone marrow-derived macrophage	increased	**MG:** increased(1), proliferation and differentiation are transcriptionally downregulated(2)<br>**SMG:** proliferation and differentiation are transcriptionally downregulated(2)	1. Armstrong JW, Gerren RA, Chapes SK. The effect of space and parabolic flight on macrophage hematopoiesis and function. Exp Cell Res. 1995;216(1):160-168. doi:10.1006/excr.1995.1020.<br>2. Shi L, Tian H, Wang P, et al. Spaceflight and simulated microgravity suppresses macrophage development via altered RAS/ERK/NFκB and metabolic pathways. Cell Mol Immunol. 2021;18(6):1489-1502. doi:10.1038/s41423-019-0346-6
IL-10	increased	**MG:** increased(1), decreased(2), no change(3)<br>**HG:** decreased(4)<br>**SMG:** increased for M1 and M2 macrophages but not M0 macrophages(5)	1. Crucian B, Stowe R, Mehta S, et al. Immune system dysregulation occurs during short duration spaceflight on board the Space Shuttle. J Clin Immunol. 2013;33(2):456-465. doi:10.1007/s10875-012-9824-7.<br>2. Crucian B, Stowe RP, Mehta S, Quiriarte H, Pierson D, Sams C. Alterations in adaptive immunity persist during long-duration spaceflight. NPJ Microgravity. 2015;1:15013. Published 2015 Sep 3. doi:10.1038/npjmgrav.2015.13<br>3. Crucian BE, Zwart SR, Mehta S, et al. Plasma cytokine concentrations indicate that in vivo hormonal regulation of immunity is altered during long-duration spaceflight. J Interferon Cytokine Res. 2014;34(10):778-786. doi:10.1089/jir.2013.0129<br>4. Jang TY, Jung AY, Kim YH. Hormetic Effect of Chronic Hypergravity in a Mouse Model of Allergic Asthma and Rhinitis. Sci Rep. 2016;6:27260. Published 2016 Jun 2. doi:10.1038/srep27260<br>5. Ludtka C, Moore E, Allen JB. The Effects of Simulated Microgravity on Macrophage Phenotype. Biomedicines. 2021;9(9):1205. Published 2021 Sep 12. doi:10.3390/biomedicines9091205
Antioxidant power	diminished	**MG:** downregulate antioxidant defense capacity(1)<br>**SMG:** reduced antioxidant power(2), reduced (total) antioxidant capacity(3,4), no change(5)	1. Hollander J, Gore M, Fiebig R, et al. Spaceflight downregulates antioxidant defense systems in rat liver. Free Radic Biol Med. 1998;24(2):385-390. doi:10.1016/s0891-5849(97)00278-5<br>2. Qu L, Chen H, Liu X, et al. Protective effects of flavonoids against oxidative stress induced by simulated microgravity in SH-SY5Y cells. Neurochem Res. 2010;35(9):1445-1454. doi:10.1007/s11064-010-0205-4<br>3. Manis C, Manca A, Murgia A, et al. Understanding the Behaviour of Human Cell Types under Simulated Microgravity Conditions: The Case of Erythrocytes. Int J Mol Sci. 2022;23(12):6876. Published 2022 Jun 20. doi:10.3390/ijms23126876<br>4. Zwart SR, Oliver SA, Fesperman JV, et al. Nutritional status assessment before, during, and after long-duration head-down bed rest. Aviat Space Environ Med. 2009;80(5 Suppl):A15-A22. doi:10.3357/asem.br07.2009<br>5. Debevec T, Pialoux V, Ehrström S, et al. FemHab: The effects of bed rest and hypoxia on oxidative stress in healthy women. J Appl Physiol (1985). 2016;120(8):930-938. doi:10.1152/japplphysiol.00919.2015

Macrophage phagocytosis	decreased	**MG:** reduced phagocytic index (1,2)<br>**SMG:** delayed phagocytosis (3)	1. Kaur I, Simons ER, Castro VA, Ott CM, Pierson DL. Changes in monocyte functions of astronauts. Brain Behav Immun. 2005;19(6):547-554. doi:10.1016/j.bbi.2004.12.006<br>2. Marcu O, Lera MP, Sanchez ME, et al. Innate immune responses of Drosophila melanogaster are altered by spaceflight. PLoS One. 2011;6(1):e15361. Published 2011 Jan 11. doi:10.1371/journal.pone.0015361<br>3. Adrian A, Schoppmann K, Sromicki J, et al. The oxidative burst reaction in mammalian cells depends on gravity. Cell Commun Signal. 2013;11:98. doi:10.1186/1478-811X-11-98.
Macrophage filopodia	decreased	/	
Stimulus-induced actin polymerization in granulocytes	decreased	/	

PROTEIN & METABOLISM

Item	Effect of ageing	Effect of altered gravity (**MG** = real microgravity, **HG** = hypergravity, **SMG** = simulated microgravity)	References
Proteostasis & proteomic	altered	**MG:** proteomics altered (1,2,3)<br>**HG:** proteomics altered (4)<br>**SMG:** proteomics altered (3), proteostasis failure(5)	1. Mao XW, Byrum S, Nishiyama NC, et al. Impact of Spaceflight and Artificial Gravity on the Mouse Retina: Biochemical and Proteomic Analysis. Int J Mol Sci. 2018;19(9):2546. Published 2018 Aug 28. doi:10.3390/ijms19092546<br>2. Brzhozovskiy A, Kononikhin A, Indeykina M, et al. Label-free study of cosmonaut's urinary proteome changes after long-duration spaceflights. Eur J Mass Spectrom (Chichester). 2017;23(4):225-229. doi:10.1177/1469066717717610<br>3. Brzhozovskiy AG, Kononikhin AS, Pastushkova LC, et al. The Effects of Spaceflight Factors on the Human Plasma Proteome, Including Both Real Space Missions and Ground-Based Experiments. Int J Mol Sci. 2019;20(13):3194. Published 2019 Jun 29. doi:10.3390/ijms20133194<br>4. Hosamani R, Leib R, Bhardwaj SR, Adams CM, Bhattacharya S. Elucidating the "Gravome": Quantitative Proteomic Profiling of the Response to Chronic Hypergravity in Drosophila. J Proteome Res. 2016;15(12):4165-4175. doi:10.1021/acs.jproteome.6b00030<br>5. Li C, Pan Y, Tan Y, Wang Y, Sun X. PINK1-Dependent Mitophagy Reduced Endothelial Hyperpermeability and Cell Migration Capacity Under Simulated Microgravity. Front Cell Dev Biol. 2022;10:896014. Published 2022 Jul 7. doi:10.3389/fcell.2022.896014
Unfold, misfold, or aggregate protein	accumulate	**MG**: reduced protein aggregates(1)<br>**SMG**: increased(2,3,4)	1. Honda Y, Higashibata A, Matsunaga Y, et al. Genes down-regulated in spaceflight are involved in the control of longevity in Caenorhabditis elegans. Sci Rep. 2012;2:487. doi:10.1038/srep00487<br>2. Li CF, Pan YK, Gao Y, Shi F, Wang YC, Sun XQ. Autophagy protects HUVECs against ER stress-mediated apoptosis under simulated microgravity. Apoptosis. 2019;24(9-10):812-825. doi:10.1007/s10495-019-01560-w<br>3. Liu LJ, Li S, Wu XT, Yang X, Sun LW. Contribution of endoplasmic reticulum stress response to the mechanosensitivity alteration in osteocytes under simulated microgravity. Acta Astronaut. 2022;191:522-527. doi:10.1016/j.actaastro.2021.11.037.<br>4. Liu P, Li D, Li W, Wang D. Mitochondrial Unfolded Protein Response to Microgravity Stress in Nematode Caenorhabditis elegans. Sci Rep. 2019;9(1):16474. Published 2019 Nov 11. doi:10.1038/s41598-019-53004-9
Heat shock protein	impaired synthesis with age and overexpression extends longevity	**MG**: reduced mrna HSP expression HSP70,HSC73,HSP47 (1), reduced mrna expression for soleus HSP70, HSP27, HSP84 (2), reduced mrna expression of HSP70,HSP90 (3)<br>**HG**: increased HSP70 (4), increased mrna HSP47 expression (5)<br>**SMG**: reduced mrna expression for soleus HSP70, HSP27, HSP84 (2), reduced mrna expression of HSP47, increased HSP70 (4)	1. Kumei Y, Morita S, Shimokawa H, et al. Inhibition of HSP70 and a collagen-specific molecular chaperone (HSP47) expression in rat osteoblasts by microgravity. Ann N Y Acad Sci. 2003;1010:476-480. doi:10.1196/annals.1299.086<br>2. Ishihara A, Fujino H, Nagatomo F, Takeda I, Ohira Y. Gene expression levels of heat shock proteins in the soleus and plantaris muscles of rats after hindlimb suspension or spaceflight. J Physiol Sci. 2008;58(6):413-417. doi:10.2170/physiolsci.RP000808<br>3. Versari S, Longinotti G, Barenghi L, Maier JA, Bradamante S. The challenging environment on board the International Space Station affects endothelial cell function by triggering oxidative stress through thioredoxin interacting protein overexpression: the ESA-SPHINX experiment. FASEB J. 2013;27(11):4466-4475. doi:10.1096/fj.13-229195<br>4. Shimada N, Moorman SJ. Changes in gravitational force cause changes in gene expression in the lens of developing zebrafish. Dev Dyn. 2006;235(10):2686-2694. doi:10.1002/dvdy.20901<br>5. Oguro A, Sakurai T, Fujita Y, et al. The molecular chaperone HSP47 rapidly senses gravitational changes in myoblasts. Genes Cells. 2006;11(11):1253-1265. doi:10.1111/j.1365-2443.2006.01021.x
Co-chaperone Hsp70	when defiencient result in accelerated ageing	/	
Heat Shock Factor 1	decreasing activity results in accelelerated ageing and overexpression extends lifespan	**SMG:** increased (1)	1. Wang C, Luo H, Zhu L, et al. Microgravity inhibition of lipopolysaccharide-induced tumor necrosis factor-α expression in macrophage cells. Inflamm Res. 2014;63(1):91-98. doi:10.1007/s00011-013-0676-2

SIRT1	upregulation improves healthy ageing	**MG:** gene upregulated (1)<br>**SMG:** decreased mrna and protein(2), decreased protein(3,4), increased mrna and protein(5)	1. Higashibata A, Hashizume T, Nemoto K, et al. Microgravity elicits reproducible alterations in cytoskeletal and metabolic gene and protein expression in space-flown Caenorhabditis elegans. NPJ Microgravity. 2016;2:15022. Published 2016 Jan 21. doi:10.1038/npjmgrav.2015.22<br>2. Liu L, Cheng Y, Wang J, et al. Simulated Microgravity Suppresses Osteogenic Differentiation of Mesenchymal Stem Cells by Inhibiting Oxidative Phosphorylation. Int J Mol Sci. 2020;21(24):9747. Published 2020 Dec 21. doi:10.3390/ijms21249747<br>3. Liu J, Peng Y, Cui Z, et al. Depressed mitochondrial biogenesis and dynamic remodeling in mouse tibialis anterior and gastrocnemius induced by 4-week hindlimb unloading. IUBMB Life. 2012;64(11):901-910. doi:10.1002/iub.1087<br>4. Chacon-Cabrera A, Gea J, Barreiro E. Short- and Long-Term Hindlimb Immobilization and Reloading: Profile of Epigenetic Events in Gastrocnemius. J Cell Physiol. 2017;232(6):1415-1427. doi:10.1002/jcp.25635<br>5. Brocca L, Rossi M, Canepari M, Bottinelli R, Pellegrino MA. Exercise Preconditioning Blunts Early Atrogenes Expression and Atrophy in Gastrocnemius Muscle of Hindlimb Unloaded Mice. Int J Mol Sci. 2021;23(1):148. Published 2021 Dec 23. doi:10.3390/ijms23010148
SIRT6	deficient results in accelelerated ageing and overexpression extends lifespan	/	
Insulin Receptor β	decreased	**SMG:** no change(1)	1. O'keefe MP, Perez FR, Kinnick TR, Tischler ME, Henriksen EJ. Development of whole-body and skeletal muscle insulin resistance after one day of hindlimb suspension. Metabolism. 2004;53(9):1215-1222. doi:10.1016/j.metabol.2004.02.025
GnRH	decreases and GnRH injection decelerates ageing	/	
Fibrinogen	increased in plasma	**MG:** increased(1), increased protein synthesis rates early flight & recovery (2), reduced fibrinogen protein synthesis rates 6 days after flight (3)<br>**SMG:** increased (4), no change (1), increased but only in women, not men (5)	1. Kuzichkin DS, Morukov BV, Markin AA, Zhuravleva OA, Zabolotskaia IV, Vostrikova LV. Fiziol Cheloveka. 2010;36(4):125-129.<br>2. Stein TP, Leskiw MJ, Schluter MD. Diet and nitrogen metabolism during spaceflight on the shuttle. J Appl Physiol (1985). 1996;81(1):82-97. doi:10.1152/jappl.1996.81.1.82<br>3. Stein TP, Schluter MD. Plasma protein synthesis after spaceflight. Aviat Space Environ Med. 2006;77(7):745-748.<br>4. Martin DG, Convertino VA, Goldwater D, Ferguson EW, Schoomaker EB. Plasma viscosity elevations with simulated weightlessness. Aviat Space Environ Med. 1986;57(5):426-431.<br>5. Pace N, Kodama AM, Price DC, Grunbaum BW, Rahlmann DF, Newsom BD. Body composition changes in men and women after 2-3 weeks of bed rest. Life Sci Space Res. 1976;14:269-274.

RBC malondialdehyde	increased	**HG:** increased during recovery(1)<br>**SMG:** increased(2,3), increased only significantly after SMG(4), decreased during SMG and increased during recovery(5)	1. Markin AA. Lipid peroxidation and the antioxidant protection system in humans exposed to hypergravity of varying intensity. Hum Physiol. 2016;42(5):546-549. doi:10.1134/S0362119716050108<br>2. Manis C, Manca A, Murgia A, et al. Understanding the Behaviour of Human Cell Types under Simulated Microgravity Conditions: The Case of Erythrocytes. Int J Mol Sci. 2022;23(12):6876. Published 2022 Jun 20. doi:10.3390/ijms23126876<br>3. Rai B, Kaur J, Catalina M, Anand SC, Jacobs R, Teughels W. Effect of simulated microgravity on salivary and serum oxidants, antioxidants, and periodontal status. J Periodontol. 2011;82(10):1478-1482. doi:10.1902/jop.2011.100711<br>4. Debevec T, Pialoux V, Ehrström S, et al. FemHab: The effects of bed rest and hypoxia on oxidative stress in healthy women. J Appl Physiol (1985). 2016;120(8):930-938. doi:10.1152/japplphysiol.00919.2015<br>5. Juravlyova OA, Markin AA, Kuzichkin DS, et al. Dynamics of oxidation stress markers during long-term antiorthostatic hypokinesia: A retrospective study. Hum Physiol. 2016;42(1):79-83. doi:10.1134/S0362119715060109.
RBC glutathione	decreased	**MG:** no change(1)<br>**SMG:** decreased (2,3), no change(4), total glutathione reduced and GSH/GSSG increased (5)	1. Buchheim JI, Matzel S, Rykova M, et al. Stress Related Shift Toward Inflammaging in Cosmonauts After Long-Duration Space Flight. Front Physiol. 2019;10:85. Published 2019 Feb 19. doi:10.3389/fphys.2019.00085<br>2. Manis C, Manca A, Murgia A, et al. Understanding the Behaviour of Human Cell Types under Simulated Microgravity Conditions: The Case of Erythrocytes. Int J Mol Sci. 2022;23(12):6876. Published 2022 Jun 20. doi:10.3390/ijms23126876<br>3. RDinarelli S, Longo G, Dietler G, et al. Erythrocyte's aging in microgravity highlights how environmental stimuli shape metabolism and morphology. Sci Rep. 2018;8:5277. doi:10.1038/s41598-018-22870-0.<br>4. Dolopikou CF, Kourtzidis IA, Tsiftsis AN, et al. Systemic redox biomarkers suggest non-redox mediated processes in the prevention of bed rest-induced muscle atrophy after exercise training: The Cologne RSL study. Acta Astronaut. 2020;168:116-122. doi:10.1016/j.actaastro.2019.12.002.<br>5. Biolo G, Di Girolamo FG, McDonnell A, et al. Effects of Hypoxia and Bed Rest on Markers of Cardiometabolic Risk: Compensatory Changes in Circulating TRAIL and Glutathione Redox Capacity. Front Physiol. 2018;9:1000. Published 2018 Jul 30. doi:10.3389/fphys.2018.01000
RBC sulphydryl	decreased	/	
RBC haemoglobin	increased	**HG:** decreases over time and decreases more for 3g than for 2g(1)<br>**SMG:** no change(2), increased(3)	1. Pecaut MJ, Miller GM, Nelson GA, Gridley DS. Hypergravity-induced immunomodulation in a rodent model: hematological and lymphocyte function analyses. J Appl Physiol (1985). 2004;97(1):29-38. doi:10.1152/japplphysiol.01304.2003<br>2. Ryan BJ, Goodrich JA, Schmidt WF, Stothard ER, Wright KP Jr, Byrnes WC. Haemoglobin mass alterations in healthy humans following four-day head-down tilt bed rest. Exp Physiol. 2016;101(5):628-640. doi:10.1113/EP085665<br>3. Lampe L, Wienhold K, Meyer G, et al. Effects of simulated microgravity (HDT) on blood fluidity. J Appl Physiol (1985). 1992;73(4):1366-1369. doi:10.1152/jappl.1992.73.4.1366
α-crystallin	decreased	SMG: no change(1)<br>HG: decreased (1)	1. Shimada N, Moorman SJ. Changes in gravitational force cause changes in gene expression in the lens of developing zebrafish. Dev Dyn. 2006;235(10):2686-2694. doi:10.1002/dvdy.20901

Sphingolipid & sphingomyelin	increased	**MG:** increased sphingolipids and sphingomyelin (1), decreased sphingolipids(2), no significant change for sphingolipids and sphingomyelin(3)<br>**HG:** no change in sphingomyelin (4)<br>**SMG:** increased sphingolipids(2), sphingomyelin decreased (5)	1. Uruno A, Saigusa D, Suzuki T, et al. Nrf2 plays a critical role in the metabolic response during and after spaceflight. Commun Biol. 2021;4(1):1381. Published 2021 Dec 9. doi:10.1038/s42003-021-02904-6
			2. Merrill AH Jr, Wang E, LaRocque R, et al. Differences in glycogen, lipids, and enzymes in livers from rats flown on COSMOS 2044. J Appl Physiol (1985). 1992;73(2 Suppl):142S-147S. doi:10.1152/jappl.1992.73.2.S142
			3. Merrill AH Jr, Wang E, Jones DP, Hargrove JL. Hepatic function in rats after spaceflight: effects on lipids, glycogen, and enzymes. Am J Physiol. 1987;252(2 Pt 2):R222-R226. doi:10.1152/ajpregu.1987.252.2.R222
			4. Albi E, Curcio F, Lazzarini A, et al. A firmer understanding of the effect of hypergravity on thyroid tissue: cholesterol and thyrotropin receptor. PLoS One. 2014;9.doi:10.1371/journal.pone.0098250.
			5. Chen ZY, Jiang N, Guo S, et al. Effect of simulated microgravity on metabolism of HGC-27 gastric cancer cells. Oncol Lett. 2020;19(5):3439-3450. doi:10.3892/ol.2020.11451
Glycerolipid	decreased	/	
SIRT3	suppressed	**SMG:** decreased(1)	1. Aventaggiato M, Barreca F, Vitiello L, et al. Role of SIRT3 in Microgravity Response: A New Player in Muscle Tissue Recovery. Cells. 2023;12(5):691. Published 2023 Feb 22. doi:10.3390/cells12050691

LYSOSOME DEGRADATION			
**Item**	**Effect of ageing**	**Effect of altered gravity** (**MG** = real microgravity, **HG** = hypergravity, **SMG** = simulated microgravity)	**References**
Lysosome-associated membrane protein 2	Autophagy-lysosomal system declines with age but with extra autophagy receptor LAMP2a no aging-associated decline in autophagic activity	**HG:** lamp-2 increases(1)	1. Smirnov AV, Mednikov DS, Shmidt MV, et al. Expression of Beclin-1 and LAMP-2 in Rat Hippocampus under Conditions of Simulated Gravity Overload in the Caudocranial Vector. Bull Exp Biol Med. 2018;165(1):105-109. doi:10.1007/s10517-018-4109-7
Polyubiquitinate proteins	accumulate	**MG:** increased mrna expression of polyubiquitin and polyubiquitinated proteins (1)<br>**SMG:** increased mrna expression of polyubiquitin and polyubiquitinated proteins (1), increased mrna expression and protein of ubiquitin ligases (2,3,4), increased expression and protein for some ubiquitin ligases and no change in others(5)	1. Ikemoto M, Nikawa T, Takeda S, et al. Space shuttle flight (STS-90) enhances degradation of rat myosin heavy chain in association with activation of ubiquitin-proteasome pathway. FASEB J. 2001;15(7):1279-1281. doi:10.1096/fj.00-0629fje<br>2. Zhang P, Li W, Liu H, et al. Dystrophin involved in the susceptibility of slow muscles to hindlimb unloading via concomitant activation of TGF-β1/Smad3 signaling and ubiquitin-proteasome degradation in mice. Cell Biochem Biophys. 2014;70(2):1057-1067. doi:10.1007/s12013-014-0023-4<br>3. Egawa T, Goto A, Ohno Y, et al. Involvement of AMPK in regulating slow-twitch muscle atrophy during hindlimb unloading in mice. Am J Physiol Endocrinol Metab. 2015;309(7):E651-E662. doi:10.1152/ajpendo.00165.2015<br>4. Shenkman BS, Belova SP, Lomonosova YN, Kostrominova TY, Nemirovskaya TL. Calpain-dependent regulation of the skeletal muscle atrophy following unloading. Arch Biochem Biophys. 2015;584:36-41. doi:10.1016/j.abb.2015.07.011<br>5. Egawa T, Kido K, Yokokawa T, et al. Involvement of receptor for advanced glycation end products in microgravity-induced skeletal muscle atrophy in mice. Acta Astronaut. 2020;176:332-340. doi:10.1016/j.actaastro.2020.07.002
Autophagy	increased activation extends lifespan	**MG:** increased(1), genes upregulated(2), genes downregulated(3)<br>**SMG:** increased autophagy (4,5)	1. Tran V, Carpo N, Cepeda C, Espinosa-Jeffrey A. Oligodendrocyte Progenitors Display Enhanced Proliferation and Autophagy after Space Flight. Biomolecules. 2023;13(2):201. Published 2023 Jan 19. doi:10.3390/biom13020201<br>2. Blaber EA, Pecaut MJ, Jonscher KR. Spaceflight Activates Autophagy Programs and the Proteasome in Mouse Liver. Int J Mol Sci. 2017;18(10):2062. Published 2017 Sep 27. doi:10.3390/ijms18102062<br>3. Vitry G, Finch R, Mcstay G, et al. Muscle atrophy phenotype gene expression during spaceflight is linked to a metabolic crosstalk in both the liver and the muscle in mice. iScience. 2022;25(10):105213. Published 2022 Sep 24. doi:10.1016/j.isci.2022.105213<br>4. Li CF, Pan YK, Gao Y, Shi F, Wang YC, Sun XQ. Autophagy protects HUVECs against ER stress-mediated apoptosis under simulated microgravity. Apoptosis. 2019;24(9-10):812-825. doi:10.1007/s10495-019-01560-w<br>5. Sambandam Y, Townsend MT, Pierce JJ, et al. Microgravity control of autophagy modulates osteoclastogenesis. Bone. 2014;61:125-131. doi:10.1016/j.bone.2014.01.004

CELL CYCLE			
**Item**	**Effect of ageing**	**Effect of altered gravity** (**MG** = real microgravity, **HG** = hypergravity, **SMG** = simulated microgravity)	**References**
PI3K	inactivation increases longevity	**HG:** activates activation (1) **SMG:** inhibit activation(1), reduced activation(2,3), increased activation(4), no change(5)	1. Dai Z, Guo F, Wu F, et al. Integrin αvβ3 mediates the synergetic regulation of core-binding factor α1 transcriptional activity by gravity and insulin-like growth factor-1 through phosphoinositide 3-kinase signaling. Bone. 2014;69:126-132. doi:10.1016/j.bone.2014.09.018<br>2. Dufour C, Holy X, Marie PJ. Skeletal unloading induces osteoblast apoptosis and targets alpha5beta1-PI3K-Bcl-2 signaling in rat bone. Exp Cell Res. 2007;313(2):394-403. doi:10.1016/j.yexcr.2006.10.021<br>3. Zhang ZK, Li J, Liu J, et al. Icaritin requires phosphatidylinositol 3 kinase (PI3K)/Akt signaling to counteract skeletal muscle atrophy following mechanical unloading. Sci Rep. 2016;6:20300. doi:10.1038/srep20300.<br>4. Zhao H, Shi Y, Qiu C, et al. Effects of Simulated Microgravity on Ultrastructure and Apoptosis of Choroidal Vascular Endothelial Cells. Front Physiol. 2021;11:577325. Published 2021 Jan 18. doi:10.3389/fphys.2020.577325<br>5. Boonyaratanakornkit JB, Cogoli A, Li CF, et al. Key gravity-sensitive signaling pathways drive T cell activation. FASEB J. 2005;19(14):2020-2022. doi:10.1096/fj.05-3778fje
PTEN	decreased expression and phosphorylation with age, and overexpression increases longevity	**SMG**: no change(1), upregulated protein and phosphorylation(2), downregulated gene(3), gene expression upregulated but protein levels reduced or non-detectable(4), gene expression slightly downregulated(5)	1. Strube F, Infanger M, Dietz C, Romswinkel A, Kraus A. Short-term effects of simulated microgravity on morphology and gene expression in human breast cancer cells. Physiol Int. 2019;106(4):311-322.<br>2. Arun RP, Sivanesan D, Vidyasekar P, et al. PTEN/FOXO3/AKT pathway regulates cell death and mediates morphogenetic differentiation of colorectal cancer cells under simulated microgravity. Sci Rep. 2017;7:5952. doi:10.1038/s41598-017-06416-4.<br>3. Ratushnyy AY, Buravkova LB. Expression of focal adhesion genes in mesenchymal stem cells under simulated microgravity. Dokl Biochem Biophys. 2017;477(1):354-356. doi:10.1134/S1607672917060035<br>4. Dietz C, Infanger M, Romswinkel A, Strube F, Kraus A. Apoptosis Induction and Alteration of Cell Adherence in Human Lung Cancer Cells under Simulated Microgravity. Int J Mol Sci. 2019;20(14):3601. Published 2019 Jul 23. doi:10.3390/ijms20143601<br>5. Ratushnyy AY, Buravkova LB. Expression of focal adhesion genes in mesenchymal stem cells under simulated microgravity. Dokl Biochem Biophys. 2017;477(1):354-356. doi:10.1134/S1607672917060035
INK4/arf locus	derepression increases with age and additional locuses extends longevity	/	
Senecent cells	increase in number	**MG:** no change in transcriptomic analyses(1) **SMG:** increased(2,3)	1. Veliz AL, Mamoun L, Hughes L, et al. Transcriptomic Effects on the Mouse Heart Following 30 Days on the International Space Station. Biomolecules. 2023;13(2):371. Published 2023 Feb 15. doi:10.3390/biom13020371<br>2. Wang J, Zhang J, Bai S, et al. Simulated microgravity promotes cellular senescence via oxidant stress in rat PC12 cells. Neurochem Int. 2009;55(7):710-716. doi:10.1016/j.neuint.2009.07.002<br>3. Takahashi H, Nakamura A, Shimizu T. Simulated microgravity accelerates aging of human skeletal muscle myoblasts at the single cell level. Biochem Biophys Res Commun. 2021;578:115-121. doi:10.1016/j.bbrc.2021.09.037
p16INK4a	increases	**SMG:** protein increased (1)	1. Wang J, Zhang J, Bai S, et al. Simulated microgravity promotes cellular senescence via oxidant stress in rat PC12 cells. Neurochem Int. 2009;55(7):710-716. doi:10.1016/j.neuint.2009.07.002

arf/ p19arf	increases	/	
p53	increases with age, elimination worsen age-related diseases	**MG:** pathway upregulated (1), activation upregulated(2), gene expression decreased(3), protein and activated form upregulated(4)<br>**SMG:** pathway upregulated(1), protein increased (5)	1. Shi L, Tian H, Wang P, et al. Spaceflight and simulated microgravity suppresses macrophage development via altered RAS/ERK/NFκB and metabolic pathways. Cell Mol Immunol. 2021;18(6):1489-1502. doi:10.1038/s41423-019-0346-6<br>2. Paulsen K, Thiel C, Timm J, et al. Microgravity-induced alterations in signal transduction in cells of the immune system. Acta Astronaut. 2010;67(9-10):1116-1125. doi:10.1016/j.actaastro.2010.06.053.<br>3. Baba T, Nishimura M, Kuwahara Y, et al. Analysis of gene and protein expression of cytochrome P450 and stress-associated molecules in rat liver after spaceflight. Pathol Int. 2008;58(9):589-595. doi:10.1111/j.1440-1827.2008.02275.x<br>4. Novoselova EG, Lunin SM, Khrenov MO, et al. Changes in immune cell signalling, apoptosis and stress response functions in mice returned from the BION-M1 mission in space. Immunobiology. 2015;220(4):500-509. doi:10.1016/j.imbio.2014.10.021<br>5. Wang J, Zhang J, Bai S, et al. Simulated microgravity promotes cellular senescence via oxidant stress in rat PC12 cells. Neurochem Int. 2009;55(7):710-716. doi:10.1016/j.neuint.2009.07.002
Regenerative potential	decreased	**SMG:** improved neural repair(1), improved wound and fracture recovery but reduced proliferation rate(2)	1. Yuge L, Sasaki A, Kawahara Y, et al. Simulated microgravity maintains the undifferentiated state and enhances the neural repair potential of bone marrow stromal cells. Stem Cells Dev. 2011;20(5):893-900. doi:10.1089/scd.2010.0294<br>2. Kong L, Wang Y, Wang H, et al. Conditioned media from endothelial progenitor cells cultured in simulated microgravity promote angiogenesis and bone fracture healing. Stem Cell Res Ther. 2021;12(1):47. Published 2021 Jan 8. doi:10.1186/s13287-020-02074-y
Hematopoietic stem cell division	decreased	**MG:** reduced cell number (1)<br>**SMG:** reduced cell number (1), faster doubling time(2), reduced cell divisions(3), increased number of BMHSC(4)	1. Wang P, Tian H, Zhang J, et al. Spaceflight/microgravity inhibits the proliferation of hematopoietic stem cells by decreasing Kit-Ras/cAMP-CREB pathway networks as evidenced by RNA-Seq assays. FASEB J. 2019;33(5):5903-5913. doi:10.1096/fj.201802413R<br>2. Nakaji-Hirabayashi T, Matsumura K, Ishihara R, et al. Enhanced proliferation and differentiation of human mesenchymal stem cells in the gravity-controlled environment. Artif Organs. 2022;46(9):1760-1770. doi:10.1111/aor.14251<br>3. Plett PA, Frankovitz SM, Abonour R, Orschell-Traycoff CM. Proliferation of human hematopoietic bone marrow cells in simulated microgravity. In Vitro Cell Dev Biol Anim. 2001;37(2):73-78. doi:10.1290/1071-2690(2001)037<0073:POHHBM>2.0.CO;2<br>4. Cao D, Song J, Ling S, et al. Hematopoietic stem cells and lineage cells undergo dynamic alterations under microgravity and recovery conditions. FASEB J. 2019;33(6):6904-6918. doi:10.1096/fj.201802421RR
CDC42	increased activity	**SMG:** increased activation(1)	1. Wang S, Yin Z, Zhao B, et al. Microgravity simulation activates Cdc42 via Rap1GDS1 to promote vascular branch morphogenesis during vasculogenesis. Stem Cell Res. 2017;25:157-165. doi:10.1016/j.scr.2017.11.002.
Senescence-associated secretory phenotype	contributes to ageing	/	

BubR1	sustained overexpression extends lifespan	/	
AUF1	premature ageing when deficient	/	
Apoptosis	increased rate	**MG:** increased(1,2), increased expression of apoptic genes(3), increased apoptosis and no change after 2 days(4)<br>**HG:** decreased expression of apoptic genes (3)<br>**SMG:** increased (5)	1. Mao XW, Byrum S, Nishiyama NC, et al. Impact of Spaceflight and Artificial Gravity on the Mouse Retina: Biochemical and Proteomic Analysis. Int J Mol Sci. 2018;19(9):2546. Published 2018 Aug 28. doi:10.3390/ijms19092546<br>2. Gridley DS, Mao XW, Tian J, et al. Genetic and Apoptotic Changes in Lungs of Mice Flown on the STS-135 Mission in Space. In Vivo. 2015;29(4):423-433.<br>3. Wehland M, Ma X, Braun M, et al. The impact of altered gravity and vibration on endothelial cells during a parabolic flight. Cell Physiol Biochem. 2013;31(2-3):432-451. doi:10.1159/000343380<br>4. Lewis ML, Reynolds JL, Cubano LA, Hatton JP, Lawless BD, Piepmeier EH. Spaceflight alters microtubules and increases apoptosis in human lymphocytes (Jurkat). FASEB J. 1998;12(11):1007-1018. doi:10.1096/fasebj.12.11.1007<br>5. Wang X, Du J, Wang D, et al. Effects of simulated microgravity on human brain nervous tissue. Neurosci Lett. 2016;627:199-204. doi:10.1016/j.neulet.2016.06.004
Cell doubling time	increased	**HG:** increased proliferation rate(1), faster doubling (2)<br>**SMG:** reduced proliferation rate(1), reduced doubling time(3,4), increased doubling time (5)	1. Huang Y, Dai ZQ, Ling SK, Zhang HY, Wan YM, Li YH. Gravity, a regulation factor in the differentiation of rat bone marrow mesenchymal stem cells. J Biomed Sci. 2009;16(1):87. Published 2009 Sep 21. doi:10.1186/1423-0127-16-87<br>2. Ciofani G, Ricotti L, Rigosa J, Menciassi A, Mattoli V, Monici M. Hypergravity effects on myoblast proliferation and differentiation. J Biosci Bioeng. 2012;113(2):258-261. doi:10.1016/j.jbiosc.2011.09.025<br>3. Nakaji-Hirabayashi T, Matsumura K, Ishihara R, et al. Enhanced proliferation and differentiation of human mesenchymal stem cells in the gravity-controlled environment. Artif Organs. 2022;46(9):1760-1770. doi:10.1111/aor.14251<br>4. Carlsson SI, Bertilaccio MT, Ballabio E, Maier JA. Endothelial stress by gravitational unloading: effects on cell growth and cytoskeletal organization. Biochim Biophys Acta. 2003;1642(3):173-179. doi:10.1016/j.bbamcr.2003.08.003<br>5. Sytkowski AJ, Davis KL. Erythroid cell growth and differentiation in vitro in the simulated microgravity environment of the NASA rotating wall vessel bioreactor. In Vitro Cell Dev Biol Anim. 2001;37(2):79-83. doi:10.1290/1071-2690(2001)037<0079:ECGADI>2.0.CO;2
GADD 153	increased	**SMG:** no change(1)	1. Hunter RB, Mitchell-Felton H, Essig DA, Kandarian SC. Expression of endoplasmic reticulum stress proteins during skeletal muscle disuse atrophy. Am J Physiol Cell Physiol. 2001;281(4):C1285-C1290. doi:10.1152/ajpcell.2001.281.4.C1285

CYTOSKELETON			
**Item**	**Effect of ageing**	**Effect of altered gravity** (**MG** = real microgravity, **HG** = hypergravity, **SMG** = simulated microgravity)	**References**
Talin	polarization response often fails in older specimens	/	
F-actin in monocyte	increases	**MG:** reduced(1)<br>**SMG:** reduced(2)	1. Meloni MA, Galleri G, Pani G, Saba A, Pippia P, Cogoli-Greuter M. Space flight affects motility and cytoskeletal structures in human monocyte cell line J-111. Cytoskeleton (Hoboken). 2011;68(2):125-137. doi:10.1002/cm.20499<br>2. Meloni MA, Galleri G, Pippia P, Cogoli-Greuter M. Cytoskeleton changes and impaired motility of monocytes at modelled low gravity. Protoplasma. 2006;229(2-4):243-249. doi:10.1007/s00709-006-0210-2
Ratio between G-actin and F-actin	shift from G-actin to F-actin	**MG:** increase of F-actin and a decrease of in G-actin(1), increase of F-actin and a decrease of in G-actin(2)<br>**HG:** increase microfilaments(1), F-actin/(F-actin+G-actin) reduced(3)<br>**SMG:** no change in microfilaments(1), decrease F-actin, increase G-actin(4)	1. Yang F, Dai Z, Tan Y, et al. Effects of altered gravity on the cytoskeleton of neonatal rat cardiocytes. Microgravity Sci Technol. 2010;22:45-52. doi:10.1007/s12217-008-9103-7.<br>2. Dai Z, Tan Y, Yang F, et al. Altered actin dynamics and functions of osteoblast-like cells in parabolic flight may involve ERK1/2. Microgravity Sci Technol. 2011;23:19-27. doi:10.1007/s12217-010-9216-7.<br>3. Lee M, Kim D, Kwon S. Hypergravity-induced changes in actin response of breast cancer cells to natural killer cells. Sci Rep. 2021;11(1):7267. Published 2021 Mar 31. doi:10.1038/s41598-021-86799-7<br>4. Meyers VE, Zayzafoon M, Douglas JT, McDonald JM. RhoA and cytoskeletal disruption mediate reduced osteoblastogenesis and enhanced adipogenesis of human mesenchymal stem cells in modeled microgravity. J Bone Miner Res. 2005;20(10):1858-1866. doi:10.1359/JBMR.050611
Vinculin	overexpression extends lifespan	**MG:** decreased expression(1,2)<br>**HG:** increased expression(3)<br>**SMG:** increased expression(4)	1. Lü D, Sun S, Zhang F, et al. Microgravity-induced hepatogenic differentiation of rBMSCs on board the SJ-10 satellite. FASEB J. 2019;33(3):4273-4286. doi:10.1096/fj.201802075R<br>2. Nassef MZ, Kopp S, Wehland M, et al. Real Microgravity Influences the Cytoskeleton and Focal Adhesions in Human Breast Cancer Cells. Int J Mol Sci. 2019;20(13):3156. Published 2019 Jun 28. doi:10.3390/ijms20133156<br>3. Kacena MA, Todd P, Gerstenfeld LC, Landis WJ. Experiments with osteoblasts cultured under hypergravity conditions. Microgravity Sci Technol. 2004;15(1):28-34. doi:10.1007/BF02870949<br>4. Chopard A, Pons F, Marini JF. Vinculin and meta-vinculin in fast and slow rat skeletal muscle before and after hindlimb suspension. Pflugers Arch. 2002;444(5):627-633. doi:10.1007/s00424-002-0872-3
Amount of actin	increased	**MG:** decreased mrna levels(1)<br>**HG:** increased(2)<br>**SMG:** decreased(3,4), decreased gene expression(5)	1. Backup P, Westerlind K, Harris S, Spelsberg T, Kline B, Turner R. Spaceflight results in reduced mRNA levels for tissue-specific proteins in the musculoskeletal system. Am J Physiol. 1994;266(4 Pt 1):E567-E573. doi:10.1152/ajpendo.1994.266.4.E567<br>2. Szulcek R, van Bezu J, Boonstra J, van Loon JJ, van Nieuw Amerongen GP. Transient Intervals of Hyper-Gravity Enhance Endothelial Barrier Integrity: Impact of Mechanical and Gravitational Forces Measured Electrically. PLoS One. 2015;10(12):e0144269. Published 2015 Dec 4. doi:10.1371/journal.pone.0144269<br>3. Shi F, Wang YC, Hu ZB, et al. Simulated Microgravity Promotes Angiogenesis through RhoA-Dependent Rearrangement of the Actin Cytoskeleton. Cell Physiol Biochem. 2017;41(1):227-238. doi:10.1159/000456060<br>4. Son H, Ho C, Le T, et al. Effects of simulated microgravity on the morphology of mouse embryonic fibroblasts (MEFs). Romanian Biotechnol Lett. 2020;25:2156-2160. doi:10.25083/rbl/25.6/2156.2160.<br>5. Giger JM, Bodell PW, Zeng M, Baldwin KM, Haddad F. Rapid muscle atrophy response to unloading: pretranslational processes involving MHC and actin. J Appl Physiol (1985). 2009;107(4):1204-1212. doi:10.1152/japplphysiol.00344.2009

Actin thickness	increased	**MG:** increased(1,2)<br>**HG:** increased(1,3,4)	1. Yang F, Dai Z, Tan Y, et al. Effects of altered gravity on the cytoskeleton of neonatal rat cardiocytes. Microgravity Sci Technol. 2010;22:45-52. doi:10.1007/s12217-008-9103-7.<br>2. Dai Z, Tan Y, Yang F, et al. Altered actin dynamics and functions of osteoblast-like cells in parabolic flight may involve ERK1/2. Microgravity Sci Technol. 2011;23:19-27. doi:10.1007/s12217-010-9216-7.<br>3. Kacena MA, Todd P, Gerstenfeld LC, Landis WJ. Experiments with osteoblasts cultured under hypergravity conditions. Microgravity Sci Technol. 2004;15(1):28-34. doi:10.1007/BF02870949<br>4. de Freitas PH, Kojima T, Ubaidus S, et al. Histological assessments on the abnormalities of mouse epiphyseal chondrocytes with short term centrifugal loading. Biomed Res. 2007;28(4):191-203. doi:10.2220/biomedres.28.191
Vimentin	expression and proteins increased	**MG:** no change in mrna expression(1), no significant change(2), increased on day 3 and decreased on day 10(3)<br>**HG:** increased(4)<br>**SMG:** no change(5)	1. Kumei Y, Morita S, Katano H, et al. Microgravity signal ensnarls cell adhesion, cytoskeleton, and matrix proteins of rat osteoblasts: osteopontin, CD44, osteonectin, and alpha-tubulin. Ann N Y Acad Sci. 2006;1090:311-317. doi:10.1196/annals.1378.034<br>2. Wuest SL, Arnold J, Gander S, et al. Microtubules and vimentin fiber stability during parabolic flights. Microgravity Sci Technol. 2020;32:921-933. doi:10.1007/s12217-020-09818-8.<br>3. Lü D, Sun S, Zhang F, et al. Microgravity-induced hepatogenic differentiation of rBMSCs on board the SJ-10 satellite. FASEB J. 2019;33(3):4273-4286. doi:10.1096/fj.201802075R<br>4. Morbidelli L, Marziliano N, Basile V, et al. Effect of hypergravity on endothelial cell function and gene expression. Microgravity Sci Technol. 2009;21:135-140. doi:10.1007/s12217-008-9067-7.<br>5. Ebnerasuly F, Hajebrahimi Z, Tabaie SM, Darbouy M. Effect of Simulated Microgravity Conditions on Differentiation of Adipose Derived Stem Cells towards Fibroblasts Using Connective Tissue Growth Factor. Iran J Biotechnol. 2017;15(4):241-251. Published 2017 Dec 29. doi:10.15171/ijb.1747
Microtubule organisation	increased percentage of abnormal organisation	**MG:** microtubule had no self-organization and locally disorganised(1), poorly defined microtubule organizing centers(2), shorter and wavier microtubules(3), altered arrangement(4)<br>**HG:** no effect (4)<br>**SMG:** altered arrangement(4), disrupted microtubule network(5)	1. Papaseit C, Pochon N, Tabony J. Microtubule self-organization is gravity-dependent. Proc Natl Acad Sci U S A. 2000;97(15):8364-8368. doi:10.1073/pnas.140029597<br>2. Lewis ML, Reynolds JL, Cubano LA, Hatton JP, Lawless BD, Piepmeier EH. Spaceflight alters microtubules and increases apoptosis in human lymphocytes (Jurkat). FASEB J. 1998;12(11):1007-1018. doi:10.1096/fasebj.12.11.1007<br>3. Nabavi N, Khandani A, Camirand A, Harrison RE. Effects of microgravity on osteoclast bone resorption and osteoblast cytoskeletal organization and adhesion. Bone. 2011;49(5):965-974. doi:10.1016/j.bone.2011.07.036<br>4. Rösner H, Wassermann T, Möller W, Hanke W. Effects of altered gravity on the actin and microtubule cytoskeleton of human SH-SY5Y neuroblastoma cells. Protoplasma. 2006;229(2-4):225-234. doi:10.1007/s00709-006-0202-2<br>5. Schatten H, Lewis ML, Chakrabarti A. Spaceflight and clinorotation cause cytoskeleton and mitochondria changes and increases in apoptosis in cultured cells. Acta Astronaut. 2001;49(3-10):399-418. doi:10.1016/s0094-5765(01)00116-3
Microtubule-associated proteins	increased	**HG:** increased microtubule-associated protein (map1a)(1)<br>**SMG:** increased map-2(2), consistent higher map-2 while in 1g it decreases with passage number(3), no effect for LC3b (4), LC3 increased(5)	1. Takumi Y, Iijima N, Suzuki N, et al. Microtubule associated protein (MAP1A) mRNA was up-regulated by hypergravity in the rat inner ear. Brain Res Mol Brain Res. 2002;108(1-2):139-142. doi:10.1016/s0169-328x(02)00517-x<br>2. Chen J, Liu R, Yang Y, et al. The simulated microgravity enhances the differentiation of mesenchymal stem cells into neurons. Neurosci Lett. 2011;505(2):171-175. doi:10.1016/j.neulet.2011.10.014<br>3. Koaykul C, Kim MH, Kawahara Y, Yuge L, Kino-Oka M. Maintenance of Neurogenic Differentiation Potential in Passaged Bone Marrow-Derived Human Mesenchymal Stem Cells Under Simulated Microgravity Conditions. Stem Cells Dev. 2019;28(23):1552-1561. doi:10.1089/scd.2019.0146<br>4. Dupré-Aucouturier S, Castells J, Freyssenet D, Desplanches D. Trichostatin A, a histone deacetylase inhibitor, modulates unloaded-induced skeletal muscle atrophy. J Appl Physiol (1985). 2015;119(4):342-351. doi:10.1152/japplphysiol.01031.2014<br>5. Yoo YM, Han TY, Kim HS. Melatonin Suppresses Autophagy Induced by Clinostat in Preosteoblast MC3T3-E1 Cells. Int J Mol Sci. 2016;17(4):526. Published 2016 Apr 8. doi:10.3390/ijms17040526
Vimentin remodelling	decreased	/	

Actin cytoskeleton remodelling rate	decreased	**MG:** increased response of actin cytoskeleton on stimulus(1)<br>**HG:** increased response of actin cytoskeleton on stimulus(1)<br>**SMG:** no effect(1), altered cytoskeleton remodelling only for very stiff substrates(2)	1. Rösner H, Wassermann T, Möller W, Hanke W. Effects of altered gravity on the actin and microtubule cytoskeleton of human SH-SY5Y neuroblastoma cells. Protoplasma. 2006;229(2-4):225-234. doi:10.1007/s00709-006-0202-2<br>2. Zhang C, Lü D, Zhang F, et al. Gravity-Vector Induces Mechanical Remodeling of rMSCs via Combined Substrate Stiffness and Orientation. Front Bioeng Biotechnol. 2022;9:724101. Published 2022 Feb 7. doi:10.3389/fbioe.2021.724101
Integrin β1	increased	**MG:** mRNA levels do not change(1), no change in distribution but the gene expression & protein is increased(2)<br>**HG:** no significant effect(2), total mRNA and protein levels not affected but increased on cell membrane(3)<br>**SMG:** increased β1 gene expression(4), decreased(5)	1. Kumei Y, Morita S, Katano H, et al. Microgravity signal ensnarls cell adhesion, cytoskeleton, and matrix proteins of rat osteoblasts: osteopontin, CD44, osteonectin, and alpha-tubulin. Ann N Y Acad Sci. 2006;1090:311-317. doi:10.1196/annals.1378.034<br>2. Aleshcheva G, Wehland M, Sahana J, et al. Moderate alterations of the cytoskeleton in human chondrocytes after short-term microgravity produced by parabolic flight maneuvers could be prevented by up-regulation of BMP-2 and SOX-9. FASEB J. 2015;29(6):2303-2314. doi:10.1096/fj.14-268151<br>3. Zhou S, Zu Y, Zhuang F, Yang C. Hypergravity-induced enrichment of β1 integrin on the cell membranes of osteoblast-like cells via caveolae-dependent endocytosis. Biochem Biophys Res Commun. 2015;463(4):928-933. doi:10.1016/j.bbrc.2015.06.037<br>4. Buken C, Sahana J, Corydon TJ, et al. Morphological and Molecular Changes in Juvenile Normal Human Fibroblasts Exposed to Simulated Microgravity. Sci Rep. 2019;9(1):11882. Published 2019 Aug 15. doi:10.1038/s41598-019-48378-9<br>5. Shi S, Li Q, Cao Q, et al. EMT Transcription Factors Are Involved in the Altered Cell Adhesion under Simulated Microgravity Effect or Overloading by Regulation of E-cadherin. Int J Mol Sci. 2020;21(4):1349. Published 2020 Feb 17. doi:10.3390/ijms21041349
Focal adhesion kinase & phosphorylation	increased	**MG:** FAK & p-FAK reduce and p-FAK was increased with intensive work-out(1), no change in FAK but reduced p-FAK(2)<br>**HG:** FAK increases when HG increases(3), increased p-FAK with time(4)<br>**SMG:** reduced p-FAK(5)	1. Rittweger J, Albracht K, Flück M, et al. Erratum: Author Correction: Sarcolab pilot study into skeletal muscle's adaptation to longterm spaceflight. NPJ Microgravity. 2018;4:23. Published 2018 Oct 24. doi:10.1038/s41526-018-0058-8<br>2. Zhang C, Li L, Jiang Y, et al. Space microgravity drives transdifferentiation of human bone marrow-derived mesenchymal stem cells from osteogenesis to adipogenesis. FASEB J. 2018;32(8):4444-4458. doi:10.1096/fj.201700208RR<br>3. Costa-Almeida R, Carvalho DTO, Ferreira MJS, et al. Continuous Exposure to Simulated Hypergravity-Induced Changes in Proliferation, Morphology, and Gene Expression of Human Tendon Cells. Stem Cells Dev. 2018;27(12):858-869. doi:10.1089/scd.2017.0206<br>4. De Cesari C, Barravecchia I, Pyankova OV, et al. Hypergravity Activates a Pro-Angiogenic Homeostatic Response by Human Capillary Endothelial Cells. Int J Mol Sci. 2020;21(7):2354. Published 2020 Mar 28. doi:10.3390/ijms21072354<br>5. Fan C, Wu Z, Cooper DML, et al. Activation of Focal Adhesion Kinase Restores Simulated Microgravity-Induced Inhibition of Osteoblast Differentiation via Wnt/B-Catenin Pathway. Int J Mol Sci. 2022;23(10):5593. Published 2022 May 17. doi:10.3390/ijms23105593
Paxillin	decreased and phosphorylation increased	**MG:** reduced paxillin(1)<br>**SMG:** no change in total paxillin but increased p-paxillin(2), paxillin increased for slow-twitch and decreased for fast-twitch muscle(3), reduced paxillin(4)	1. Nabavi N, Khandani A, Camirand A, Harrison RE. Effects of microgravity on osteoclast bone resorption and osteoblast cytoskeletal organization and adhesion. Bone. 2011;49(5):965-974. doi:10.1016/j.bone.2011.07.036<br>2. Koaykul C, Kim MH, Kawahara Y, Yuge L, Kino-Oka M. Alterations in Nuclear Lamina and the Cytoskeleton of Bone Marrow-Derived Human Mesenchymal Stem Cells Cultured Under Simulated Microgravity Conditions. Stem Cells Dev. 2019;28(17):1167-1176. doi:10.1089/scd.2018.0229<br>3. Gordon SE, Flück M, Booth FW. Selected Contribution: Skeletal muscle focal adhesion kinase, paxillin, and serum response factor are loading dependent. J Appl Physiol (1985). 2001;90(3):1174-1165. doi:10.1152/jappl.2001.90.3.1174<br>4. Shi S, Li Q, Cao Q, et al. EMT Transcription Factors Are Involved in the Altered Cell Adhesion under Simulated Microgravity Effect or Overloading by Regulation of E-cadherin. Int J Mol Sci. 2020;21(4):1349. Published 2020 Feb 17. doi:10.3390/ijms21041349

RAC1	increased activation	**SMG:** increased activity(1), decreased activity(2), increased for upside down cells(3)	1. Wang S, Yin Z, Zhao B, et al. Microgravity simulation activates Cdc42 via Rap1GDS1 to promote vascular branch morphogenesis during vasculogenesis. Stem Cell Res. 2017;25:157-165. doi:10.1016/j.scr.2017.11.002.<br>2. Tan X, Xu A, Zhao T, et al. Simulated microgravity inhibits cell focal adhesions leading to reduced melanoma cell proliferation and metastasis via FAK/RhoA-regulated mTORC1 and AMPK pathways. Sci Rep. 2018;8:3769. doi:10.1038/s41598-018-20459-1.<br>3. Ju Z, Thomas TN, Chiu YJ, et al. Adaptation and Changes in Actin Dynamics and Cell Motility as Early Responses of Cultured Mammalian Cells to Altered Gravitational Vector. Int J Mol Sci. 2022;23(11):6127. Published 2022 May 30. doi:10.3390/ijms23116127
Paxillin,Vinculin and FAK location	located more in perinuclear region instead of the cell surface	**MG:** vinculin close to the cell membrane as globular clusters (1) **HG:** vinculin concentrated on the cell perimeter(2), FAK located mainly in perinuclear location(3) **SMG:** paxillin is more located near the leading edges(4)	1. Meloni MA, Galleri G, Pani G, Saba A, Pippia P, Cogoli-Greuter M. Space flight affects motility and cytoskeletal structures in human monocyte cell line J-111. Cytoskeleton (Hoboken). 2011;68(2):125-137. doi:10.1002/cm.20499<br>2. Kacena MA, Todd P, Gerstenfeld LC, Landis WJ. Experiments with osteoblasts cultured under hypergravity conditions. Microgravity Sci Technol. 2004;15(1):28-34. doi:10.1007/BF02870949<br>3. Costa-Almeida R, Carvalho DTO, Ferreira MJS, et al. Continuous Exposure to Simulated Hypergravity-Induced Changes in Proliferation, Morphology, and Gene Expression of Human Tendon Cells. Stem Cells Dev. 2018;27(12):858-869. doi:10.1089/scd.2017.0206<br>4. Koaykul C, Kim MH, Kawahara Y, Yuge L, Kino-Oka M. Alterations in Nuclear Lamina and the Cytoskeleton of Bone Marrow-Derived Human Mesenchymal Stem Cells Cultured Under Simulated Microgravity Conditions. Stem Cells Dev. 2019;28(17):1167-1176. doi:10.1089/scd.2018.0229
MRTF-A	decreased	/	
RhoA	increased	**MG:** decreased for neonatal and it seems increased for adult cells(1) **HG:** increased activation(2) **SMG:** decreased(3,4), decreased activity(5)	1. Baio J, Martinez AF, Silva I, et al. Cardiovascular progenitor cells cultured aboard the International Space Station exhibit altered developmental and functional properties. NPJ Microgravity. 2018;4:13. Published 2018 Jul 26. doi:10.1038/s41526-018-0048-x<br>2. Koyama T, Kimura C, Hayashi M, Watanabe M, Karashima Y, Oike M. Hypergravity induces ATP release and actin reorganization via tyrosine phosphorylation and RhoA activation in bovine endothelial cells. Pflugers Arch. 2009;457(4):711-719. doi:10.1007/s00424-008-0544-z<br>3. Tan X, Xu A, Zhao T, et al. Simulated microgravity inhibits cell focal adhesions leading to reduced melanoma cell proliferation and metastasis via FAK/RhoA-regulated mTORC1 and AMPK pathways. Sci Rep. 2018;8:3769. doi:10.1038/s41598-018-20459-1.<br>4. Shi F, Wang YC, Hu ZB, et al. Simulated Microgravity Promotes Angiogenesis through RhoA-Dependent Rearrangement of the Actin Cytoskeleton. Cell Physiol Biochem. 2017;41(1):227-238. doi:10.1159/000456060<br>5. Wan Q, Cho E, Yokota H, Na S. RhoA GTPase interacts with beta-catenin signaling in clinorotated osteoblasts. J Bone Miner Metab. 2013;31(5):520-532. doi:10.1007/s00774-013-0449-6
Actin cap	disorganized or completely eliminated	/	

Tubulin	protein decreases in fibroblasts and increases in the heart	**MG:** decreased β-tubulin(1), increased α-tubilin(2), decreased α-tubilin mrna levels(3), no significant effect on α-tubilin(4)<br>**HG:** tubilin proteins are downregulated(5)	1. Meloni MA, Galleri G, Pani G, Saba A, Pippia P, Cogoli-Greuter M. Space flight affects motility and cytoskeletal structures in human monocyte cell line J-111. Cytoskeleton (Hoboken). 2011;68(2):125-137. doi:10.1002/cm.20499<br>2. Paulsen K, Tauber S, Goelz N, et al. Severe disruption of the cytoskeleton and immunologically relevant surface molecules in a human macrophageal cell line in microgravity—Results of an in vitro experiment on board of the Shenzhou-8 space mission. Acta Astronaut. 2014;94(1):277-292. doi:10.1016/j.actaastro.2013.06.007.<br>3. Kumei Y, Morita S, Katano H, et al. Microgravity signal ensnarls cell adhesion, cytoskeleton, and matrix proteins of rat osteoblasts: osteopontin, CD44, osteonectin, and alpha-tubulin. Ann N Y Acad Sci. 2006;1090:311-317. doi:10.1196/annals.1378.034<br>4. Zhang Y, Lu T, Wong M, et al. Transient gene and microRNA expression profile changes of confluent human fibroblast cells in spaceflight. FASEB J. 2016;30(6):2211-2224. doi:10.1096/fj.201500121<br>5. Wehland M, Ma X, Braun M, et al. The impact of altered gravity and vibration on endothelial cells during a parabolic flight. Cell Physiol Biochem. 2013;31(2-3):432-451. doi:10.1159/000343380
α5 integrin	increased	**SMG:** decreased in first days and no difference after 7 days(1), increased(2)	1. Dufour C, Holy X, Marie PJ. Skeletal unloading induces osteoblast apoptosis and targets alpha5beta1-PI3K-Bcl-2 signaling in rat bone. Exp Cell Res. 2007;313(2):394-403. doi:10.1016/j.yexcr.2006.10.021<br>2. Nelson LJ, Walker SW, Hayes PC, Plevris JN. Low-shear modelled microgravity environment maintains morphology and differentiated functionality of primary porcine hepatocyte cultures. Cells Tissues Organs. 2010;192(2):125-140. doi:10.1159/000308893
Akt phosphorylation	increased	**MG:** for masseter increased and for tibialis anterior decreased(1)<br>**HG:** increased for tibialis anterior and no change for soleus(2)<br>**SMG:** no change(3), decreased(4,5)	1. Philippou A, Minozzo FC, Spinazzola JM, et al. Masticatory muscles of mouse do not undergo atrophy in space. FASEB J. 2015;29(7):2769-2779. doi:10.1096/fj.14-267336<br>2. Mirzoev T, Tyganov S, Petrova I, et al. Divergent anabolic signalling responses of murine soleus and tibialis anterior muscles to chronic 2G hypergravity. Sci Rep. 2017;7:3514. doi:10.1038/s41598-017-03758-x.<br>3. Fuentes TI, Appleby N, Raya M, et al. Simulated Microgravity Exerts an Age-Dependent Effect on the Differentiation of Cardiovascular Progenitors Isolated from the Human Heart. PLoS One. 2015;10(7):e0132378. Published 2015 Jul 10. doi:10.1371/journal.pone.0132378<br>4. Baek MO, Ahn CB, Cho HJ, et al. Simulated microgravity inhibits C2C12 myogenesis via phospholipase D2-induced Akt/FOXO1 regulation. Sci Rep. 2019;9:14910. doi:10.1038/s41598-019-51410-7<br>5. Sakuma K, Watanabe K, Hotta N, et al. The adaptive responses in several mediators linked with hypertrophy and atrophy of skeletal muscle after lower limb unloading in humans. Acta Physiol (Oxf). 2009;197(2):151-159. doi:10.1111/j.1748-1716.2009.01995.x
Desmin in cardiac cells	intact desmin decreased	**MG:** no change(236)	1. Ogneva IV, Maximova MV, Larina IM. Structure of cortical cytoskeleton in fibers of mouse muscle cells after being exposed to a 30-day space flight on board the BION-M1 biosatellite. J Appl Physiol (1985). 2014;116(10):1315-1323. doi:10.1152/japplphysiol.00134.2014

EXTRACELLULAR MATRIX			
**Item**	**Effect of ageing**	**Effect of altered gravity** (**MG** = real microgravity, **HG** = hypergravity, **SMG** = simulated microgravity)	**References**
Matrix metalloproteinase	increased	**MG:** MMP 1,3, 10 gene expression increases(1) **HG:** MMP1 and 2 are upregulated(2) **SMG:** MMP 1, 3 gene expression increase(3), MMP 9 unchanged after 7 days but decreased after 14 days (4), MMP 8 and 9 increase(5)	1. Blaber EA, Dvorochkin N, Lee C, et al. Microgravity induces pelvic bone loss through osteoclastic activity, osteocytic osteolysis, and osteoblastic cell cycle inhibition by CDKN1a/p21. PLoS One. 2013;8(4):e61372. Published 2013 Apr 18. doi:10.1371/journal.pone.0061372
			2. De Cesari C, Barravecchia I, Pyankova OV, et al. Hypergravity Activates a Pro-Angiogenic Homeostatic Response by Human Capillary Endothelial Cells. Int J Mol Sci. 2020;21(7):2354. Published 2020 Mar 28. doi:10.3390/ijms21072354
			3. Buken C, Sahana J, Corydon TJ, et al. Morphological and Molecular Changes in Juvenile Normal Human Fibroblasts Exposed to Simulated Microgravity. Sci Rep. 2019;9(1):11882. Published 2019 Aug 15. doi:10.1038/s41598-019-48378-9
			4. Visigalli D, Strangio A, Palmieri D, Manduca P. Hind limb unloading of mice modulates gene expression at the protein and mRNA level in mesenchymal bone cells. BMC Musculoskelet Disord. 2010;11:147. Published 2010 Jul 5. doi:10.1186/1471-2474-11-147
			5. Rai B, Kaur J, Catalina M. Bone mineral density, bone mineral content, gingival crevicular fluid (matrix metalloproteinases, cathepsin K, osteocalcin), and salivary and serum osteocalcin levels in human mandible and alveolar bone under conditions of simulated microgravity. J Oral Sci. 2010;52(3):385-390. doi:10.2334/josnusd.52.385
Collagen fibrils	more fragmentation	**MG:** decreased/ fragmented(1), decreased collagen fibril size(2), no change(3,4) **HG:** disrupted and wavy collagen fibrils(5) **SMG:** no change in collagen fibril size or width(3)	1. Blottner D, Moriggi M, Trautmann G, et al. Space Omics and Tissue Response in Astronaut Skeletal Muscle after Short and Long Duration Missions. Int J Mol Sci. 2023;24(4):4095. Published 2023 Feb 17. doi:10.3390/ijms24044095
			2. Deymier AC, Schwartz AG, Lim C, et al. Multiscale effects of spaceflight on murine tendon and bone. Bone. 2020;131:115152. doi:10.1016/j.bone.2019.115152
			3. Montufar-Solis D, Duke PJ, Durnova G. Spaceflight and age affect tibial epiphyseal growth plate histomorphometry. J Appl Physiol (1985). 1992;73(2 Suppl):19S-25S. doi:10.1152/jappl.1992.73.2.S19
			4. Katsuda S, Yamasaki M, Waki H, et al. Spaceflight affects postnatal development of the aortic wall in rats. Biomed Res Int. 2014;2014:490428. doi:10.1155/2014/490428
			5. de Freitas PH, Kojima T, Ubaidus S, et al. Histological assessments on the abnormalities of mouse epiphyseal chondrocytes with short term centrifugal loading. Biomed Res. 2007;28(4):191-203. doi:10.2220/biomedres.28.191
Ratio of collagen to elastin in aorta	increased	**MG:** no change (1,2)	1. Sofronova SI, Tarasova OS, Gaynullina D, et al. Spaceflight on the Bion-M1 biosatellite alters cerebral artery vasomotor and mechanical properties in mice [published correction appears in J Appl Physiol (1985). 2016 May 15;120(10):1267. doi: 10.1152/japplphysiol.zdg-1838.corr.2016]. J Appl Physiol (1985). 2015;118(7):830-838. doi:10.1152/japplphysiol.00976.2014
			2. Katsuda S, Yamasaki M, Waki H, et al. Spaceflight affects postnatal development of the aortic wall in rats. Biomed Res Int. 2014;2014:490428. doi:10.1155/2014/490428
Collagen fibre tortuosity	decreased	/	
Fibronectin	increased	**MG:** first few hours reduced synthesis and protein and after ~1 day no change(1), increased on day 4 and no change on day 5(2), no change(3) **HG:** decreased(4), no change(5)	1. Hughes-Fulford M, Gilbertson V. Osteoblast fibronectin mRNA, protein synthesis, and matrix are unchanged after exposure to microgravity. FASEB J. 1999;13 Suppl:S121-S127. doi:10.1096/fasebj.13.9001.s121
			2. Kumei Y, Morita S, Katano H, et al. Microgravity signal ensnarls cell adhesion, cytoskeleton, and matrix proteins of rat osteoblasts: osteopontin, CD44, osteonectin, and alpha-tubulin. Ann N Y Acad Sci. 2006;1090:311-317. doi:10.1196/annals.1378.034
			3. Zhang Y, Lu T, Wong M, et al. Transient gene and microRNA expression profile changes of confluent human fibroblast cells in spaceflight. FASEB J. 2016;30(6):2211-2224. doi:10.1096/fj.201500121
			4. Kacena MA, Todd P, Gerstenfeld LC, Landis WJ. Experiments with osteoblasts cultured under hypergravity conditions. Microgravity Sci Technol. 2004;15(1):28-34. doi:10.1007/BF02870949
			5. Vitry G, Finch R, Mcstay G, et al. Muscle atrophy phenotype gene expression during spaceflight is linked to a metabolic crosstalk in both the liver and the muscle in mice. iScience. 2022;25(10):105213. Published 2022 Sep 24. doi:10.1016/j.isci.2022.105213

Procollagen	production decreased	**MG:** increased newly synthetized procollagen(1)	1. Neutelings T, Nusgens BV, Liu Y, et al. Skin physiology in microgravity: a 3-month stay aboard ISS induces dermal atrophy and affects cutaneous muscle and hair follicles cycling in mice. NPJ Microgravity. 2015;1:15002. Published 2015 May 27. doi:10.1038/npjmgrav.2015.2
Advanced glycation end products crosslinks	increased	**SMG:** increased cancellous bone and no change for cortical bone (1), increased in trabecular bone but no significant changes in cortical bone(2), increased in blood plasma(3)	1. Liu CJ, Yang X, Wang SH, et al. Preventing Disused Bone Loss through Inhibition of Advanced Glycation End Products. Int J Mol Sci. 2023;24(5):4953. Published 2023 Mar 3. doi:10.3390/ijms24054953<br>2. Liu CJ, Yang X, Mao Y, et al. The alteration of advanced glycation end products and its potential role on bone loss under microgravity. Acta Astronaut. 2023;206:114-122. doi:10.1016/j.actaastro.2023.02.019<br>3. Egawa T, Kido K, Yokokawa T, Fujibayashi M, Goto K, Hayashi T. Involvement of receptor for advanced glycation end products in microgravity-induced skeletal muscle atrophy in mice. Acta Astronaut. 2020;176:332-340. doi:10.1016/j.actaastro.2020.07.002
Disaccharide from heparan sulfate	altered	/	
Collagen in cardiac ECM	increased	**MG**: decreased in the heart and downregulated collagen maturation(1)	1. Walls S, Diop S, Birse R, et al. Prolonged Exposure to Microgravity Reduces Cardiac Contractility and Initiates Remodeling in Drosophila. Cell Rep. 2020;33(10):108445. doi:10.1016/j.celrep.2020.108445
Tropoelastin	decreased	**SMG:** gene expression is upreguated(1)	1. Ebnerasuly F, Hajebrahimi Z, Tabaie SM, Darbouy M. Effect of Simulated Microgravity Conditions on Differentiation of Adipose Derived Stem Cells towards Fibroblasts Using Connective Tissue Growth Factor. Iran J Biotechnol. 2017;15(4):241-251. Published 2017 Dec 29. doi:10.15171/ijb.1747

CELL MECHANICS			
**Item**	**Effect of ageing**	**Effect of altered gravity** (**MG** = real microgravity, **HG** = hypergravity, **SMG** = simulated microgravity)	**References**
Skeletal muscle stiffness	increased	**MG:** decreased in medial gastrocnemius type II but not Soleus type I(1), no change in soleus and increased for activated and not activated in tibialis anterior(2)<br>**HG:** increased(3)<br>**SMG:** in relaxed state stiffness of the sarcolemmais reduced, in activated state it depends on the muscle(4), reduced for soleus (5)	1. Fitts RH, Desplanches D, Romatowski JG, Widrick JJ. Spaceflight effects on single skeletal muscle fiber function in the rhesus monkey. Am J Physiol Regul Integr Comp Physiol. 2000;279(5):R1546-R1557. doi:10.1152/ajpregu.2000.279.5.R1546<br>2. Ogneva IV, Maximova MV, Larina IM. Structure of cortical cytoskeleton in fibers of mouse muscle cells after being exposed to a 30-day space flight on board the BION-M1 biosatellite. J Appl Physiol (1985). 2014;116(10):1315-1323. doi:10.1152/japplphysiol.00134.2014<br>3. Ogneva IV, Gnyubkin V, Laroche N, Maximova MV, Larina IM, Vico L. Structure of the cortical cytoskeleton in fibers of postural muscles and cardiomyocytes of mice after 30-day 2-g centrifugation. J Appl Physiol (1985). 2015;118(5):613-623. doi:10.1152/japplphysiol.00812.2014<br>4. Ogneva IV. Transversal stiffness of fibers and desmin content in leg muscles of rats under gravitational unloading of various durations. J Appl Physiol (1985). 2010;109(6):1702-1709. doi:10.1152/japplphysiol.00793.2010<br>5. Ogneva IV. Transversal stiffness and beta-actin and alpha-actinin-4 content of the M. soleus fibers in the conditions of a 3-day reloading after 14-day gravitational unloading. J Biomed Biotechnol. 2011;2011:393405. doi:10.1155/2011/393405
Cell stiffness	increased	**MG:** no change(1)<br>**HG:** increased(2)<br>**SMG:** decreased(3,4), increased(5)	1. Ogneva IV, Maximova MV, Larina IM. Structure of cortical cytoskeleton in fibers of mouse muscle cells after being exposed to a 30-day space flight on board the BION-M1 biosatellite. J Appl Physiol (1985). 2014;116(10):1315-1323. doi:10.1152/japplphysiol.00134.2014<br>2. Ogneva IV, Gnyubkin V, Laroche N, Maximova MV, Larina IM, Vico L. Structure of the cortical cytoskeleton in fibers of postural muscles and cardiomyocytes of mice after 30-day 2-g centrifugation. J Appl Physiol (1985). 2015;118(5):613-623. doi:10.1152/japplphysiol.00812.2014<br>3. Janmaleki M, Pachenari M, Seyedpour SM, Shahghadami R, Sanati-Nezhad A. Impact of Simulated Microgravity on Cytoskeleton and Viscoelastic Properties of Endothelial Cell. Sci Rep. 2016;6:32418. Published 2016 Sep 1. doi:10.1038/srep32418<br>4. Wubshet NH, Arreguin-Martinez E, Nail M, et al. Simulating microgravity using a random positioning machine for inducing cellular responses to mechanotransduction in human osteoblasts. Rev Sci Instrum. 2021;92(11):114101. doi:10.1063/5.0056366<br>5. Mao X, Chen Z, Luo Q, Zhang B, Song G. Simulated microgravity inhibits the migration of mesenchymal stem cells by remodeling actin cytoskeleton and increasing cell stiffness. Cytotechnology. 2016;68(6):2235-2243. doi:10.1007/s10616-016-0007-x
Cell migration and motility	Slower, shorter distance and lower directionality	**MG:** no change for lung cells(1)<br>**HG:** increased for directed movement and for low serum in total movement(2),<br>**SMG:** decreased directed movement for low serum(2), decreased migration speed and decreased number of migrated fibroblasts(3), decreased migration(4), reduced monocyte migration and reduced distance(5)	1. Montgomery PO Jr, Cook JE, Reynolds RC, et al. The response of single human cells to zero gravity. In Vitro. 1978;14(2):165-173. doi:10.1007/BF02618218<br>2. Sanford GL, Harris-Hooker S, Lui J, Bosah FN. Wound healing following injury to vascular smooth muscle cell cultures is modulated by culture under hypergravity. J Gravit Physiol. 1999;6(1):P29-P30.<br>3. Cialdai F, Colciago A, Pantalone D, et al. Effect of Unloading Condition on the Healing Process and Effectiveness of Platelet Rich Plasma as a Countermeasure: Study on In Vivo and In Vitro Wound Healing Models. Int J Mol Sci. 2020;21(2):407. Published 2020 Jan 9. doi:10.3390/ijms21020407<br>4. Cialdai F, Vignali L, Morbidelli L, et al. Modeled microgravity affects fibroblast functions related to wound healing. Microgravity Sci Technol. 2017;29:121-132. doi:10.1007/s12217-016-9532-7.<br>5. Meloni MA, Galleri G, Pippia P, Cogoli-Greuter M. Cytoskeleton changes and impaired motility of monocytes at modelled low gravity. Protoplasma. 2006;229(2-4):243-249. doi:10.1007/s00709-006-0210-2
Tenocyte density, cellularity and shape	decreased density and cellularity and elongated shape	/	

Cell size, shape and spreading	decreased spreading, decreased size and more round for fibroblasts, and increased size for endothelial cells	**MG:** more contracted cells(1), increased size for endothelial cells(2), increased size(3)<br>**HG:** more fibroblast spreading and microspikes(4)<br>**SMG:** reduced cell area(5)	1. Meloni MA, Galleri G, Pani G, Saba A, Pippia P, Cogoli-Greuter M. Space flight affects motility and cytoskeletal structures in human monocyte cell line J-111. Cytoskeleton (Hoboken). 2011;68(2):125-137. doi:10.1002/cm.20499<br>2. Kapitonova MY, Kuznetsov SL, Froemming GR, et al. Effects of space mission factors on the morphology and function of endothelial cells. Bull Exp Biol Med. 2013;154(6):796-801. doi:10.1007/s10517-013-2059-7<br>3. Kapitonova MY, Kuznetsov SL, Salim N, et al. Morphological and phenotypical characteristics of human osteoblasts after short-term space mission. Bull Exp Biol Med. 2014;156(3):393-398. doi:10.1007/s10517-014-2357-8<br>4. Croute F, Gaubin Y, Pianezzi B, Soleilhavoup JP. Effects of hypergravity on the morphology, the cytoskeleton, the synthesis of extracellular macromolecules and the activity of degradative enzymes. Microgravity Sci Technol. 1994;366:31.<br>5. Son H, Ho C, Le T, et al. Effects of simulated microgravity on the morphology of mouse embryonic fibroblasts (MEFs). Romanian Biotechnol Lett. 2020;25:2156-2160. doi:10.25083/rbl/25.6/2156.2160
Keratinocyte outgrowth	decreased	**SMG:** enhanced keratinocyte migration(1)	1. Ranieri D, Proietti S, Dinicola S, et al. Simulated microgravity triggers epithelial mesenchymal transition in human keratinocytes. Sci Rep. 2017;7(1):538. Published 2017 Apr 3. doi:10.1038/s41598-017-00602-0
Membrane viscosity	decreased	**MG:** increased after landing and decreased the following days(1)<br>**HG:** increasing viscosity with increasing gravity level(2)	1. Grigoriev AI, Maksimov GV, Morukov BV, et al. Investigation of erythrocyte shape, plasma membrane fluidity and conformation of haemoglobin haemoporphyrin under the influence of long-term space flight. J Gravit Physiol. 2004;11(2):P79-P80.<br>2. Woodcock EM, Girvan P, Eckert J, et al. Measuring Intracellular Viscosity in Conditions of Hypergravity. Biophys J. 2019;116(10):1984-1993. doi:10.1016/j.bpj.2019.03.038
Nucleus stiffness	increased	/	
Fibroblast adhesion	decreased	**HG:** cell-cell decreased & cell substrate increased for fibroblasts and increased both for neoplastic fibroblast(1)<br>**SMG:** cell-cell and cell-substrate increased for fibroblast but decreased for neoplastic fibroblast (1), decreased adhesion to substrate(2), increased adhesion to Cu+ Hyal surface(3)	1. Pippia P, Meloni MA, Cossu G, Cogoli-Greuter M, Cogoli A. Cellular adhesion in neoplastic and syngeneic normal cells under altered gravitational conditions. J Gravit Physiol. 1998;5(1):P165-P166.<br>2. Cialdai F, Vignali L, Morbidelli L, et al. Modeled microgravity affects fibroblast functions related to wound healing. Microgravity Sci Technol. 2017;29:121-132. doi:10.1007/s12217-016-9532-7<br>3. Monici M, Cialdai F, Romano G, et al. An in vitro study on tissue repair: impact of unloading on cells involved in the remodelling phase. Microgravity Sci Technol. 2011;23:391-401. doi:10.1007/s12217-011-9259-4
Anisotropy in skeletal muscle	decreased	/	

Lamellipodium extension	decreased	**MG:** reduced pseudopodia(1), formation of lamellipodia and filopodia that are more pronounced with time(2)<br>**Combined MG &HG:** increase of pseudopoidia and lamellipodia (3), increased lamellipodia extension activity (4)<br>**HG:** increased lamellipodia extension activity (4)<br>**SMG:** no change(4), increased lamellipodia (5)	1. Meloni MA, Galleri G, Pani G, Saba A, Pippia P, Cogoli-Greuter M. Space flight affects motility and cytoskeletal structures in human monocyte cell line J-111. Cytoskeleton (Hoboken). 2011;68(2):125-137. doi:10.1002/cm.20499<br>2. Nassef MZ, Kopp S, Wehland M, et al. Real Microgravity Influences the Cytoskeleton and Focal Adhesions in Human Breast Cancer Cells. Int J Mol Sci. 2019;20(13):3156. Published 2019 Jun 28. doi:10.3390/ijms20133156<br>3. Schulz H, Dietrichs D, Wehland M, et al. In Prostate Cancer Cells Cytokines Are Early Responders to Gravitational Changes Occurring in Parabolic Flights. Int J Mol Sci. 2022;23(14):7876. Published 2022 Jul 17. doi:10.3390/ijms23147876<br>4. Rösner H, Wassermann T, Möller W, Hanke W. Effects of altered gravity on the actin and microtubule cytoskeleton of human SH-SY5Y neuroblastoma cells. Protoplasma. 2006;229(2-4):225-234. doi:10.1007/s00709-006-0202-2<br>5. Siamwala JH, Reddy SH, Majumder S, et al. Simulated microgravity perturbs actin polymerization to promote nitric oxide-associated migration in human immortalized Eahy926 cells. Protoplasma. 2010;242(1-4):3-12. doi:10.1007/s00709-010-0114-z
Wound healing	delayed	**MG:** impaired wound healing(1), no change(2)<br>**HG:** delayed wound closure(3)<br>**SMG:** reduced wound closure(4), delayed wound healing (5)	1. Davidson JM, Aquino AM, Woodward SC, Wilfinger WW. Sustained microgravity reduces intrinsic wound healing and growth factor responses in the rat. FASEB J. 1999;13(2):325-329. doi:10.1096/fasebj.13.2.325<br>2. Stauber WT, Fritz VK, Burkovskaya TE, Ilyina-Kakueva EI. Effect of spaceflight on the extracellular matrix of skeletal muscle after a crush injury. J Appl Physiol (1985). 1992;73(2 Suppl):74S-81S. doi:10.1152/jappl.1992.73.2.S74<br>3. Radstake WE, Gautam K, Miranda S, et al. Gravitational effects on fibroblasts' function in relation to wound healing. NPJ Microgravity. 2023;9(1):48. Published 2023 Jun 21. doi:10.1038/s41526-023-00286-z<br>4. Cialdai F, Vignali L, Morbidelli L, et al. Modeled microgravity affects fibroblast functions related to wound healing. Microgravity Sci Technol. 2017;29:121-132. doi:10.1007/s12217-016-9532-7<br>5. Cialdai F, Colciago A, Pantalone D, Rizzo AM, Zava S, Morbidelli L, Celotti F, Bani D, Monici M. Effect of Unloading Condition on the Healing Process and Effectiveness of Platelet Rich Plasma as a Countermeasure: Study on In Vivo and In Vitro Wound Healing Models. International Journal of Molecular Sciences. 2020; 21(2):407. https://doi.org/10.3390/ijms21020407
Outgrowth endothelial cell	decreased	/	
Blood plasma viscosity	Linearly increase with age	**SMG:** decreased after SMG(1)	1. Lampe L, Wienhold K, Meyer G, et al. Effects of simulated microgravity (HDT) on blood fluidity. J Appl Physiol (1985). 1992;73(4):1366-1369. doi:10.1152/jappl.1992.73.4.1366
RBC volume	increased	**MG:** mean corpuscular volume RBC increased(1)<br>**HG:** mean corpuscular volume RBC decreased(2)	1. Kunz H, Quiriarte H, Simpson RJ, et al. Alterations in hematologic indices during long-duration spaceflight. BMC Hematol. 2017;17:12. Published 2017 Sep 8. doi:10.1186/s12878-017-0083-y<br>2. Pecaut MJ, Miller GM, Nelson GA, Gridley DS. Hypergravity-induced immunomodulation in a rodent model: hematological and lymphocyte function analyses. J Appl Physiol (1985). 2004;97(1):29-38. doi:10.1152/japplphysiol.01304.2003
RBC deformability	decreased	**HG:** decreased but not significantly(1)<br>**SMG:** reduced deformability(2,3)	1. Marijke G, Vera A, Tobias V, Wilhelm B, Stefan S. Erythrocyte deformability and aggregation responses to intermittent and continuous artificial gravity exposure. Life Sci Space Res (Amst). 2017;12:61-66. doi:10.1016/j.lssr.2017.01.004<br>2. Lampe L, Wienhold K, Meyer G, et al. Effects of simulated microgravity (HDT) on blood fluidity. J Appl Physiol (1985). 1992;73(4):1366-1369. doi:10.1152/jappl.1992.73.4.1366<br>3. Shen X, Dong Q, Chen J, et al. Erythrocyte deformation in simulated weightless human and rabbits. J Gravit Physiol. 1997;4(3):61-65.

RBC surface/volume ratio	increased	/	
RBC viscoelastic time constant	decreases	/	
Actin filament sliding speed	decreased	/	
osteoblasts responsiveness to fluid flow	decreased	/	
Osteoblasts intracellular calcium	decreased basal [Ca2+] activity	**MG:** no change(1)<br>**SMG:** decreased intracellular calcium concentration (2), increased after 24 hours and no change again after 48 hours(3)	1. Klopp E, Graff D, Struckmeier J, et al. The osteoblast mechano-receptor, microgravity perception and thermodynamics. J Gravit Physiol. 2002;9(1):P269-P270.<br>2. Sun Z, Li Y, Zhou H, et al. Simulated microgravity reduces intracellular-free calcium concentration by inhibiting calcium channels in primary mouse osteoblasts. J Cell Biochem. 2019;120(3):4009-4020. doi:10.1002/jcb.27685<br>3. Morabito C, Guarnieri S, Cucina A, Bizzarri M, Mariggiò MA. Antioxidant Strategy to Prevent Simulated Microgravity-Induced Effects on Bone Osteoblasts. Int J Mol Sci. 2020;21(10):3638. Published 2020 May 21. doi:10.3390/ijms21103638
Stimulus-induced differentiation of MPPs	decreased mechano-response to substrate stiffness	/	
Mechanosensitivity of osteocytes	decreased	**HG:** increased response to IGF-1(1)<br>**SMG:** osteocytes become less mechanosensitive, decreased percentage of responsive cells and show decreased calcium intensity and spikes and require higher ca2+ initiation time(2), decreased mechanosenitivity(3), reduced response to IGF-1(1), reduced number of osteocytes (4)	1. Dai Z, Guo F, Wu F, et al. Integrin αvβ3 mediates the synergetic regulation of core-binding factor α1 transcriptional activity by gravity and insulin-like growth factor-1 through phosphoinositide 3-kinase signaling. Bone. 2014;69:126-132. doi:10.1016/j.bone.2014.09.018<br>2. Liu X, Yan Z, Cai J, et al. Glucose- and glutamine-dependent bioenergetics sensitize bone mechanoresponse after unloading by modulating osteocyte calcium dynamics. J Clin Invest. 2023;133(3):e164508. Published 2023 Feb 1. doi:10.1172/JCI164508<br>3. Liu LJ, Li S, Wu XT, Yang X, Sun LW. Contribution of endoplasmic reticulum stress response to the mechanosensitivity alteration in osteocytes under simulated microgravity. Acta Astronaut. 2022;191:522-527. doi:10.1016/j.actaastro.2021.11.037<br>4. Metzger CE, Brezicha JE, Elizondo JP, Narayanan SA, Hogan HA, Bloomfield SA. Differential responses of mechanosensitive osteocyte proteins in fore- and hindlimbs of hindlimb-unloaded rats. Bone. 2017;105:26-34. doi:10.1016/j.bone.2017.08.002

RBC aggregation	increased	**HG:** increases for constant g but not intermediate(1), increased but quickly lowers again after 30 min(2)<br>**SMG:** increased aggregation(3)	1. Marijke G, Vera A, Tobias V, Wilhelm B, Stefan S. Erythrocyte deformability and aggregation responses to intermittent and continuous artificial gravity exposure. Life Sci Space Res (Amst). 2017;12:61-66. doi:10.1016/j.lssr.2017.01.004<br>2. Limper U, Ahnert T, Maegele M, et al. Simulated Hypergravity Activates Hemostasis in Healthy Volunteers. J Am Heart Assoc. 2020;9(24):e016479. doi:10.1161/JAHA.120.016479<br>3. Lampe L, Wienhold K, Meyer G, et al. Effects of simulated microgravity (HDT) on blood fluidity. J Appl Physiol (1985). 1992;73(4):1366-1369. doi:10.1152/jappl.1992.73.4.1366
Cell traction forces	increased	**HG:** decreased for up to 5.4g and an increased for higher gravity fields up to 19.5 g(1)	1. Kumei Y, Nakamura H, Morita S, et al. Space flight and insulin-like growth factor-I signaling in rat osteoblasts. Ann N Y Acad Sci. 2002;973:75-78. doi:10.1111/j.1749-6632.2002.tb04609.x
Membrane ruffling	decreased	/	

CELL SIGNALING

Item	Effect of ageing	Effect of altered gravity (**MG** = real microgravity, **HG** = hypergravity, **SMG** = simulated microgravity)	References
Insulin-like Growth Factor 1	levels decline	**MG:** decreased mrna levels(1), decreased plasma IGF-1 concentration(2), reduced in soleus, increased in edl (3), increased mrna levels in tibiae(3) **SMG:** increased mrna levels in tibiae (3),reduced(4)	1. Kumei Y, Nakamura H, Morita S, et al. Space flight and insulin-like growth factor-I signaling in rat osteoblasts. Ann N Y Acad Sci. 2002;973:75-78. doi:10.1111/j.1749-6632.2002.tb04609.x<br>2. Adams GR, McCue SA, Bodell PW, Zeng M, Baldwin KM. Effects of spaceflight and thyroid deficiency on hindlimb development. I. Muscle mass and IGF-I expression. J Appl Physiol (1985). 2000;88(3):894-903. doi:10.1152/jappl.2000.88.3.894<br>3. Bikle DD, Harris J, Halloran BP, Morey-Holton E. Altered skeletal pattern of gene expression in response to spaceflight and hindlimb elevation. Am J Physiol. 1994;267(6 Pt 1):E822-E827. doi:10.1152/ajpendo.1994.267.6.E822<br>4. Lawler JM, Kwak HB, Kim JH, Lee Y, Hord JM, Martinez DA. Biphasic stress response in the soleus during reloading after hind limb unloading. Med Sci Sports Exerc. 2012;44(4):600-609. doi:10.1249/MSS.0b013e31823ab37a
p38 Mitogen-Activated Protein Kinases	activation increased	**MG:** increased p38 MAPK activation/ more phosphorylation (1), no significant change (2) **SMG:** reduced activation (3), increased activation(4,5)	1. Zhang C, Li L, Jiang Y, et al. Space microgravity drives transdifferentiation of human bone marrow-derived mesenchymal stem cells from osteogenesis to adipogenesis. FASEB J. 2018;32(8):4444-4458. doi:10.1096/fj.201700208RR<br>2. Verhaar AP, Hoekstra E, Tjon AS, et al. Dichotomal effect of space flight-associated microgravity on stress-activated protein kinases in innate immunity. Sci Rep. 2014;4:5468. Published 2014 Jun 27. doi:10.1038/srep05468<br>3. Wang J, Han C, Lu Z, et al. Simulated microgravity suppresses MAPK pathway-mediated innate immune response to bacterial infection and induces gut microbiota dysbiosis. FASEB J. 2020;34(11):14631-14644. doi:10.1096/fj.202001428R<br>4. Wang C, Chen H, Luo H, et al. Microgravity activates p38 MAPK-C/EBPβ pathway to regulate the expression of arginase and inflammatory cytokines in macrophages. Inflamm Res. 2015;64(5):303-311. doi:10.1007/s00011-015-0811-3<br>5. Lin SC, Gou GH, Hsia CW, et al. Simulated Microgravity Disrupts Cytoskeleton Organization and Increases Apoptosis of Rat Neural Crest Stem Cells Via Upregulating CXCR4 Expression and RhoA-ROCK1-p38 MAPK-p53 Signaling. Stem Cells Dev. 2016;25(15):1172-1193. doi:10.1089/scd.2016.0040
Phosphorylated p38 location	less in nucleus	**MG:** no significant change(1) **SMG:** phosphorylated p38 located only in the nucleus(2)	1. Verhaar AP, Hoekstra E, Tjon AS, et al. Dichotomal effect of space flight-associated microgravity on stress-activated protein kinases in innate immunity. Sci Rep. 2014;4:5468. Published 2014 Jun 27. doi:10.1038/srep05468<br>2. Lin SC, Gou GH, Hsia CW, et al. Simulated Microgravity Disrupts Cytoskeleton Organization and Increases Apoptosis of Rat Neural Crest Stem Cells Via Upregulating CXCR4 Expression and RhoA-ROCK1-p38 MAPK-p53 Signaling. Stem Cells Dev. 2016;25(15):1172-1193. doi:10.1089/scd.2016.0040
Secreted Frizzled Related Protein 1	increased	/	
Wnt signalling	increased	**MG:** pathway genes downregulated (1), suppression of canonical Wnt signaling in neonatal CPCs and the promotion in adult CPC(2) **HG:** pathway inhibitors downregulated or no change(1) **SMG:** Wnt/β-catenin pathway inhibitors upregulated(1), surpressed (3), downregulated (4), altered with 2 Wnt signaling genes upregulated and 2 downregulated and mechanosensitivity of Wnt signaling adversly altered for all genes (5)	1. Yamamoto T, Ikegame M, Furusawa Y, et al. Osteoclastic and Osteoblastic Responses to Hypergravity and Microgravity: Analysis Using Goldfish Scales as a Bone Model. Zoolog Sci. 2022;39(4):10.2108/zs210107. doi:10.2108/zs210107<br>2. Baio J, Martinez AF, Silva I, et al. Cardiovascular progenitor cells cultured aboard the International Space Station exhibit altered developmental and functional properties. NPJ Microgravity. 2018;4:13. Published 2018 Jul 26. doi:10.1038/s41526-018-0048-x<br>3. Cheng Y, Zhou Y, Lv W, Luo Q, Song G. Simulated Microgravity Inhibits Rodent Dermal Fibroblastic Differentiation of Mesenchymal Stem Cells by Suppressing ERK/β-Catenin Signaling Pathway. Int J Mol Sci. 2021;22(19):10702. Published 2021 Oct 2. doi:10.3390/ijms221910702<br>4. Fan C, Wu Z, Cooper DML, et al. Activation of Focal Adhesion Kinase Restores Simulated Microgravity-Induced Inhibition of Osteoblast Differentiation via Wnt/B-Catenin Pathway. Int J Mol Sci. 2022;23(10):5593. Published 2022 May 17. doi:10.3390/ijms23105593<br>5. Yang X, Sun LW, Wu XT, Liang M, Fan YB. Impact of shear stress and simulated microgravity on osteocytes using a new rotation cell culture device. Acta Astronaut. 2015;116:286-298. doi:10.1016/j.actaastro.2015.07.020

Caveolin-1	increased	**MG:** increased expression(1,2), reduced but not significant(3), **HG:**no change(1), increased caveolin-1 expression(3) **SMG:** decreased expression(4), increased expression(5)	1. Aleshcheva G, Wehland M, Sahana J, et al. Moderate alterations of the cytoskeleton in human chondrocytes after short-term microgravity produced by parabolic flight maneuvers could be prevented by up-regulation of BMP-2 and SOX-9. FASEB J. 2015;29(6):2303-2314. doi:10.1096/fj.14-268151<br>2. Masini MA, Albi E, Barmo C, et al. The impact of long-term exposure to space environment on adult mammalian organisms: a study on mouse thyroid and testis. PLoS One. 2012;7(4):e35418. doi:10.1371/journal.pone.0035418<br>3. Kopp S, Krüger M, Feldmann S, et al. Thyroid cancer cells in space during the TEXUS-53 sounding rocket mission – The THYROID Project. Sci Rep. 2018;8:10355. doi:10.1038/s41598-018-28695-1.<br>4. Shi F, Zhao TZ, Wang YC, et al. The Impact of Simulated Weightlessness on Endothelium-Dependent Angiogenesis and the Role of Caveolae/Caveolin-1. Cell Physiol Biochem. 2016;38(2):502-513. doi:10.1159/000438646<br>5. Grenon SM, Jeanne M, Aguado-Zuniga J, Conte MS, Hughes-Fulford M. Effects of gravitational mechanical unloading in endothelial cells: association between caveolins, inflammation and adhesion molecules. Sci Rep. 2013;3:1494. doi:10.1038/srep01494
mTORC1	inhibition or downregulating extends lifespan and longevity	**HG:** no change for soleus and increased for tibialis for MTORC1 related partners(1) **SMG:** downregulated MTORC1 related partners(2,3,4)	1. Mirzoev T, Tyganov S, Petrova I, et al. Divergent anabolic signalling responses of murine soleus and tibialis anterior muscles to chronic 2G hypergravity. Sci Rep. 2017;7:3514. doi:10.1038/s41598-017-03758-x.<br>2. Zhao T, Li R, Tan X, et al. Simulated Microgravity Reduces Focal Adhesions and Alters Cytoskeleton and Nuclear Positioning Leading to Enhanced Apoptosis via Suppressing FAK/RhoA-Mediated mTORC1/NF-κB and ERK1/2 Pathways. Int J Mol Sci. 2018;19(7):1994. Published 2018 Jul 8. doi:10.3390/ijms19071994<br>3. Tan X, Xu A, Zhao T, et al. Simulated microgravity inhibits cell focal adhesions leading to reduced melanoma cell proliferation and metastasis via FAK/RhoA-regulated mTORC1 and AMPK pathways. Sci Rep. 2018;8:3769. doi:10.1038/s41598-018-20459-1.<br>4. Mirzoev T, Tyganov S, Vilchinskaya N, Lomonosova Y, Shenkman B. Key Markers of mTORC1-Dependent and mTORC1-Independent Signaling Pathways Regulating Protein Synthesis in Rat Soleus Muscle During Early Stages of Hindlimb Unloading. Cell Physiol Biochem. 2016;39(3):1011-1020. doi:10.1159/000447808
mTORC2	low levels of mTORC1 but normal levels of mTORC2 increase lifespan	**SMG**:decreased the level of mSIN1 in mTORC2 (1), decreased Rictor in mTORC2 (2)	1. Baek MO, Ahn CB, Cho HJ, et al. Simulated microgravity inhibits C2C12 myogenesis via phospholipase D2-induced Akt/FOXO1 regulation. Sci Rep. 2019;9:14910. doi:10.1038/s41598-019-51410-7<br>2. Zhao T, Li R, Tan X, et al. Simulated Microgravity Reduces Focal Adhesions and Alters Cytoskeleton and Nuclear Positioning Leading to Enhanced Apoptosis via Suppressing FAK/RhoA-Mediated mTORC1/NF-κB and ERK1/2 Pathways. Int J Mol Sci. 2018;19(7):1994. Published 2018 Jul 8. doi:10.3390/ijms19071994
NAD+	decreases with age	**HG:** reduced(1) **SMG:** reduced levels(2)	1. Slenzka K, Appel R, Rahmann H. Metabolic adaptation to long term changes in gravity environment. Adv Space Res. 1998;22(2):273-276. doi:10.1016/s0273-1177(98)80019-7<br>2. Huang Y, Dou Y, Yang B, et al. Nicotinamide mononucleotide supplementation mitigates osteopenia induced by modeled microgravity in rats. Cell Stress Chaperones. 2023;28(4):385-394. doi:10.1007/s12192-023-01356-7
Phosphorylated mTOR & protein	decreased	**SMG:** p-mTOR decreased (1,2), p-mTOR increased (3), reduced total mTOR protein(4), no change in p-mTOR first week but increased after 14 days and total protein increased after 7 and 14 days(5)	1. Yoo YM, Han TY, Kim HS. Melatonin Suppresses Autophagy Induced by Clinostat in Preosteoblast MC3T3-E1 Cells. Int J Mol Sci. 2016;17(4):526. Published 2016 Apr 8. doi:10.3390/ijms17040526<br>2. Dupont E, Cieniewski-Bernard C, Bastide B, Stevens L. Electrostimulation during hindlimb unloading modulates PI3K-AKT downstream targets without preventing soleus atrophy and restores slow phenotype through ERK. Am J Physiol Regul Integr Comp Physiol. 2011;300(2):R408-R417. doi:10.1152/ajpregu.00793.2009<br>3. Yoo YM, Park JH, Seo DH, et al. Activation of mTOR for the loss of skeletal muscle in a hindlimb-suspended rat model. Int J Precis Eng Manuf. 2015;16:1003-1010. doi:10.1007/s12541-015-0130-1<br>4. Jespersen JG, Mikkelsen UR, Nedergaard A, et al. Alterations in molecular muscle mass regulators after 8 days immobilizing Special Forces mission. Scand J Med Sci Sports. 2015;25(2):175-183. doi:10.1111/sms.12170<br>5. Liu H, Blough ER, Arvapalli R, et al. Regulation of contractile proteins and protein translational signaling in disused muscle. Cell Physiol Biochem. 2012;30(5):1202-1214. doi:10.1159/000343310
Osteocyte dendrite number and connectivity	decreased	**MG:** no change in dendrite number, but more brances and altered morphology(1,2) **SMG:** reduced dendrite in osteocyte(3)	1. Laranjeiro R, Harinath G, Pollard AK, et al. Spaceflight affects neuronal morphology and alters transcellular degradation of neuronal debris in adult Caenorhabditis elegans. iScience. 2021;24(2):102105. Published 2021 Jan 29. doi:10.1016/j.isci.2021.102105<br>2. Mikheeva I, Mikhailova G, Shtanchaev R, Arkhipov V, Pavlik L. Influence of a 30-day spaceflight on the structure of motoneurons of the trochlear nerve nucleus in mice. Brain Res. 2021;1758:147331. doi:10.1016/j.brainres.2021.147331<br>3. Xu H, Wu J, Weng Y, Zhang J, Shang P. Two-dimensional clinorotation influences cellular morphology, cytoskeleton and secretion of MLO-Y4 osteocyte-like cells. Biologia. 2012;67. doi:10.2478/s11756-011-0161-8
